# Stromal inflammation is a targetable driver of hematopoietic aging

**DOI:** 10.1038/s41556-022-01053-0

**Published:** 2023-01-17

**Authors:** Carl A. Mitchell, Evgenia V. Verovskaya, Fernando J. Calero-Nieto, Oakley C. Olson, James W. Swann, Xiaonan Wang, Aurélie Hérault, Paul V. Dellorusso, Si Yi Zhang, Arthur Flohr Svendsen, Eric M. Pietras, Sietske T. Bakker, Theodore T. Ho, Berthold Göttgens, Emmanuelle Passegué

**Affiliations:** 1 Columbia Stem Cell Initiative, Department of Genetics and Development, Columbia University Irving Medical Center, New York, New York 10032, USA; 2 The Eli and Edythe Broad Center of Regeneration Medicine and Stem Cell Research, Department of Medicine, Division Hematology/Oncology, University of California San Francisco, San Francisco, California 94143, USA; 3 Wellcome and MRC Cambridge Stem Cell Institute, Department of Haematology, Cambridge University, Jeffrey Cheah Biomedical Centre Puddicombe Way, Cambridge CB2 0AW, UK

## Abstract

Hematopoietic aging is marked by a loss of regenerative capacity and skewed differentiation from hematopoietic stem cells (HSC) leading to impaired blood production. Signals from the bone marrow (BM) niche tailor blood production, but the contribution of the old niche to hematopoietic aging remains unclear. Here, we characterize the inflammatory milieu that drives both niche and hematopoietic remodeling. We find decreased numbers and functionality of osteoprogenitors (OPr) at the endosteum and expansion of central marrow LepR^+^ mesenchymal stromal cells (MSC-L) associated with deterioration of the sinusoidal vasculature, which together create a degraded and inflamed old BM niche. Niche inflammation, in turn, drives chronic activation of emergency myelopoiesis pathways in old HSCs and multipotent progenitors (MPP), which promotes myeloid differentiation at the expense of lymphoid and erythroid commitment and hinders hematopoietic regeneration. Moreover, we show how production of IL-1β by the damaged endosteum acts in trans to drive the proinflammatory nature of the central marrow with damaging consequences for the old blood system. Remarkably, niche deterioration, HSC dysfunction, and defective regeneration can all be ameliorated by blocking IL-1 signaling. Our results demonstrate that targeting IL-1 as a key mediator of niche inflammation is a tractable strategy to improve blood production during aging.

Hematopoietic function severely declines with age, causing anemia, impaired adaptive immunity, cancer and autoimmune disease in the elderly^1^. Aging also leads to major changes in bones and the bone marrow (BM) cavity, which together provide specialized niches for hematopoietic stem and progenitor cells (HSPC)^2–5^. Blood production is tailored in part by differential production of distinct hematopoietic stem cells (HSC)-derived multipotent progenitor (MPP) subsets with specific myeloid (MPP2, MPP3) or lymphoid (MPP4) lineage biases, which in turn give rise to different lineage-restricted progenitors and mature cells^6,7^. Niche cells and secreted factors, in particular pro-inflammatory cytokines, play important roles in controlling blood output by regulating HSC behavior, directing the production of progenitor cells, modulating stress hematopoiesis, and playing context-dependent roles in leukemic transformation^2,3,7,8^. Circulating levels of pro-inflammatory cytokines increase over time and contribute to the stereotypical decline in tissue integrity with age^9,10^. By mid-life (~ 50 years in humans/~ 12 months in mice), bones themselves are already old with thinning, impaired fracture repair, and reduced endocrine function^11^. In contrast, the most salient features of hematopoietic aging are not fully pronounced until later in life (~ 70 years in humans/~ 20-24 months in mice) and include an expansion of dysfunctional HSCs with reduced engraftment potential and blood production capability^4^. It is now well appreciated that cell-intrinsic features such as replication stress, epigenetic remodeling and metabolic rewiring are important drivers of old HSC dysfunction, with such hardwired features not reverted by systemic rejuvenation interventions^12^. It is also clear that niche degradation contributes to old HSC dysfunction^4,5^, but much less is known about the mechanisms underlying the crosstalk between the old niche and old blood system.

The BM niche is comprised of many cell types including mesenchymal stromal cells (MSC) and their osteolineage cell (OLC) derivatives like osteoprogenitor (OPr) cells, endothelial cells (EC), the sympathetic nervous system, adipocytes, and specific mature blood cells such as megakaryocytes (Mk) and macrophages^2,3^. Components of the BM microenvironment have now been extensively catalogued by imaging and molecular profiling at single cell resolution^13–16^. The marrow cavity is also partitioned between the endosteum lining the inner bone surface and the central marrow perivascular space, with each location exhibiting distinct cellular compositions and functions in regulating hematopoiesis^2–4^. Recent studies have described important age-related changes in individual niche components, particularly in the vasculature^17,18^, innervation^19,20^ and specific stromal populations^21,22^, and reported heightened inflammation in the aged marrow cavity^20,23,25^. However, the precise molecular underpinnings of niche aging have yet to be fully understood. Here, we comprehensively dissect the aged BM niche to determine its contribution to hematopoietic aging and investigate stromal-derived interleukin-1 (IL-1) as a key factor that can be targeted for functional anti-aging interventions.

## Aging remodels the BM niche

**Extended Data Figure 1:**
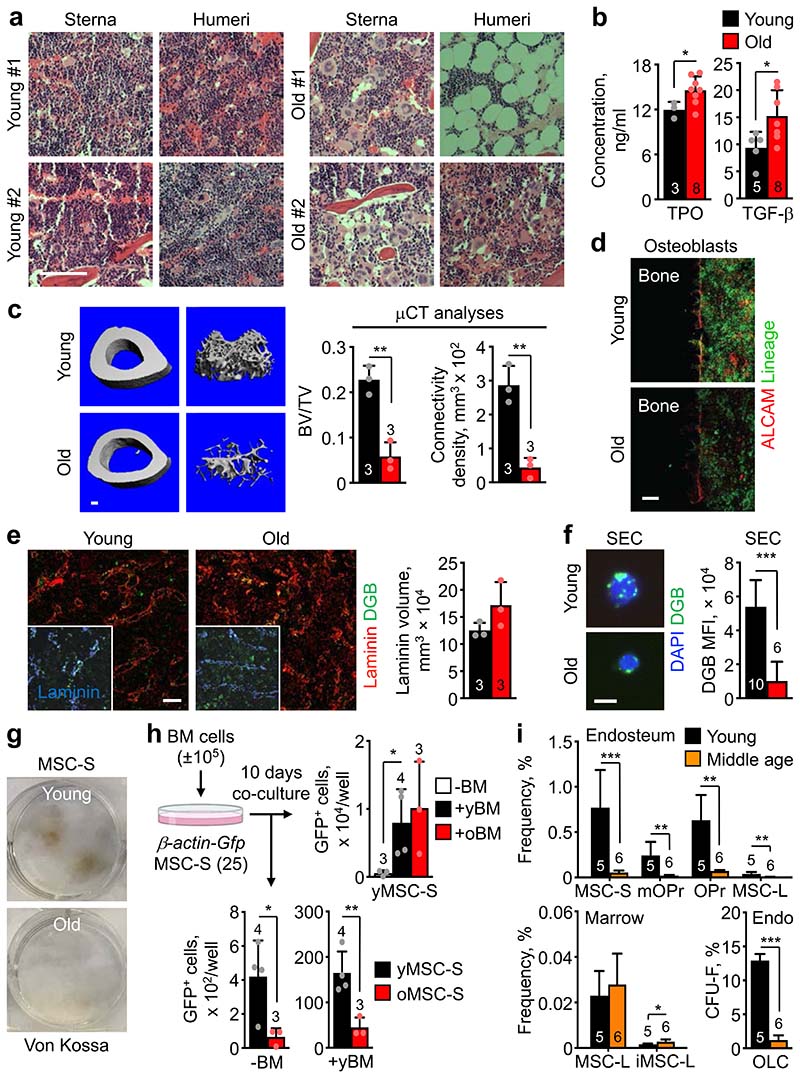
Gross analysis of the remodeled old BM cavity. **a**, H&E staining of humeri and sterna of 2 individual young and old mice. Scale bar, 100 µm. **b**, TPO and TGF-b levels in young and old BM fluids. Results are from 2 independent cohorts. **c**, µCT analyses of young and old tibias with representative images of cortical and trabecular regions (left) and quantification of bone volume/total volume (BV/TV) and connectivity density (right). **d**, Representative image of bone lining ALCAM^+^ osteoblasts immunofluorescence staining in young and old mice. Scale bar, 100 µm. **e**, Representative images and quantification of immunofluorescence staining of vascular volume (laminin) and vascular leakage by dragon-green beads (DGB) diffusion assay in young and old BM. Scale bar, 50 µm. **f**, Representative images and quantification by flow cytometry of DGB endocytosis in young and old marrow SEC; scale bar, 5 µm. Results are from 3 independent cohorts. **g**, Representative images of Von Kossa staining in young and old endosteal MSC-S. **h**, Experimental scheme for the indicated co-culture experiments showing the effects of young or old BM cells on young MSC-S (top right), and young BM cells on young and old MSC-S (bottom). **i**, Frequency of endosteal and marrow mesenchymal populations in young and middle age (13-month-old) mice with changes in CFU-F from endosteal OLCs (bottom right). Data are means ± S.D; *p ≤ 0.05, **p ≤ 0.01, ***p ≤ 0.001.

We first surveyed bone structure and composition in cohorts of young (~ 2 months old) and old (~ 24 months old) C57BL/6 wild type (WT) mice. Hematoxylin and eosin (H&E) staining showed a fully penetrant accumulation of Mks in old mice that was accompanied by elevated TGF-β and TPO levels in old BM fluids (Extended Data Fig. 1a,b), consistent with the Mk bias of old HSCs^26,27^. In contrast, we only observed infrequent accumulation of adipocytes, and only in certain bones, suggesting that aging in mice is not consistently associated with increased marrow adipogenesis as in humans. Micro-computed tomography (μCT) analyses confirmed the decay of trabecular and cortical bones in old mice^28^, which corresponded to a reduction in bone-lining ALCAM^+^ osteoblasts (Extended Data Fig. 1c,d). These results confirm the stereotypical changes associated with aging in ~ 24-month-old WT mice.

**Figure 1:**
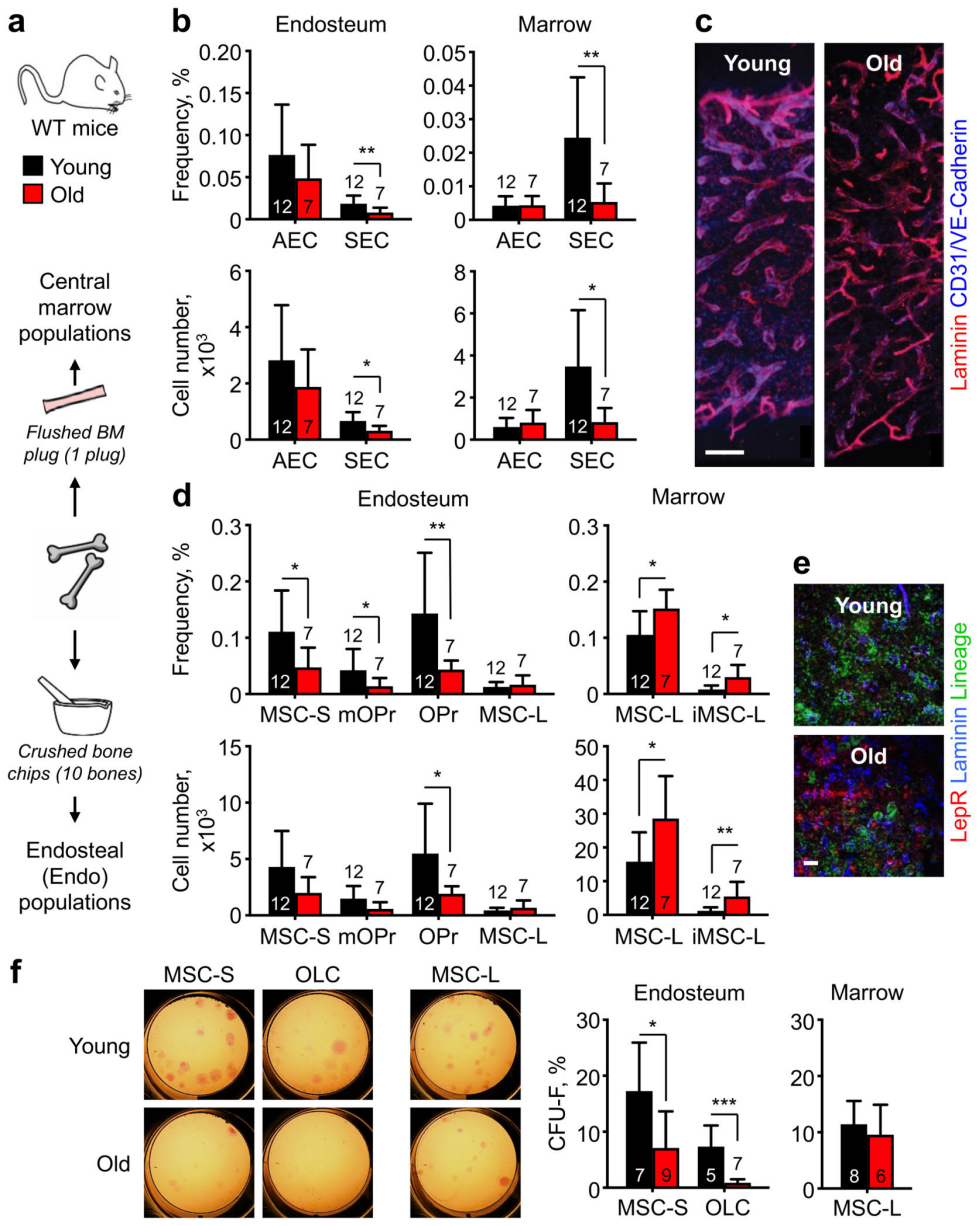
Remodeled BM microenvironment with age. **a**, Experimental scheme to investigate endosteal (Endo) and central marrow (Marrow) stromal populations in young (~ 8 week of age) and old (~ 24 months of age) C57BL/6 wild type (WT) mice. Results are from 6 independently analyzed groups of 1 or 2 young or old mice. Cellularity data per mouse are for 1 BM plug for marrow populations and bone chips from 10 bones for endosteal populations. **b**, Changes in endosteal and marrow endothelial cell (EC) populations with age showing frequency (top) and total cell numbers (bottom). AEC, arteriolar endothelial cell; SEC, sinusoidal endothelial cell. **c**, Whole mount staining of the BM vasculature in young and old mice. Scale bar, 100 µm. **d**, Changes in endosteal and marrow mesenchymal populations with age showing frequency (top) and total cell numbers (bottom). MSC, Sca-1^+^ peri-arteriolar mesenchymal stroma cell; mOPr, PDGFRα^+^ multipotent osteoprogenitor; OPr, PDGFRα^-^ osteoprogenitor; MSC-L, LepR^+^ peri-sinusoidal mesenchymal stroma cell; iMSC-L, Sca-1^low^ inflammatory MSC-L. **e**, Representative image of MSC-L immunofluorescence staining in young and old BM. Scale bar, 38 µm. **f**, Representative pictures and quantification of fibroblast colony-forming units (CFU-F) from young and old MSC-S, osteoblastic lineage cell (OLC) and MSC-L. Results are from 5 independent cohorts. Data are means ± S.D.; *p ≤ 0.05, **p ≤ 0.01, ***p ≤ 0.001.

To gain a comprehensive understanding of BM niche degradation with age, we next employed a refined version of our flow cytometric method^29^ to investigate in the same animal both endosteal niche populations using bone chips, and central marrow niche populations using flushed BM plugs (Fig. 1a; Supplementary Information SI.1a). Regarding the vasculature, while the overall frequency, number and preferential endosteal location of CD31^hi^/Sca-1^hi^ arteriolar ECs (AEC) was not significantly affected with age, we observed a significant reduction in CD31^lo^/Sca-1^lo^ sinusoidal EC (SEC) frequency and numbers both in the central marrow and at the endosteum in old mice (Fig, 1b). This contrasts with previous high definition imaging^17^ and our own whole mount immunofluorescence analyses showing enhanced branching and dysmorphia of the old central marrow sinusoidal network (Fig. 1c), which indicate poor recovery of old SECs for flow analyses and suggest fragilization with age. Moreover, we confirmed unchanged vascular volume^17,18^ and found leakage of injected Dragon Green beads (DGB) into the BM cavity with age, which was associated with decreased DGB endocytosis in old SECs (Extended Data Fig. 1e,f), consistent with vascular damage, increased leakiness and SEC dysfunction rather than increased active transcytosis. Accordingly, age-related changes in vascular stiffness and BM hypoxia have been shown to alter vascular integrity and contribute to degraded endothelial integrity^30^.

Regarding the mesenchymal populations, old mice showed a decreased frequency of endosteal periarteriolar Sca-1^+^ MSCs (MSC-S) and their OLC derivatives, which can be separated between multipotent PDGFRα^+^ OPr (mOPr) and more committed PDGFRα^-^ OPr (Fig. 1d). Conversely, in the central marrow, we found an increase in perisinusoidal LepR^+^ MSCs (MSC-L) frequency and the striking emergence of an inflammatory Sca-1^int^ MSC-L subset (iMSC-L) (Fig. 1d,e). Similar iMSC-L cells were observed following poly (I:C) treatment in young mice (Supplementary Information SI.1b), confirming the ability of some MSC-L to up-regulate Sca-1 upon inflammatory exposure. Functional assessment revealed loss of fibroblastic colony-forming unit (CFU-F) potential in old endosteal MSC-S and OLC populations, but no change in old central marrow MSC-L CFU-F potential (Fig. 1f), in line with recent findings that age-related impairment of osteogenesis originates in endosteal MSCs^31^. This is further supported by reduced calcium deposition from old MSC-S grown in osteogenic differentiation medium (Extended Data Fig. 1g). The functional decline of old MSC-S appeared to be cell-intrinsic since both young and old BM cells could similarly stimulate young MSC-S colony formation, but young BM cells could not rescue the defective growth of old MSC-S in co-culture experiments (Extended Data Fig. 1h). The changes in endosteal populations were already observed in 13-month-old middle-aged mice (Extended Data Fig. 1i), consistent with human aging where bone degradation precedes hematopoietic aging^11^. Collectively, these results reveal a profound remodeling of the old niche, with numerical loss and functional deterioration of endosteal mesenchymal populations, and expansion of inflammatory MSC-L alongside dysmorphia and fragilization of the sinusoidal network in the central marrow. They also indicate that the expanded MSC-L compartment does not compensate for age-related bone loss, likely due to their known bias towards adipogenesis at the expense of osteogenesis^32,33^ and the loss with age of an osteogenic subset of osteolectin-expressing periarteriolar MSC-L^22^.

## Abnormal inflammatory signaling in old BM niche cells

**Extended Data Figure 2:**
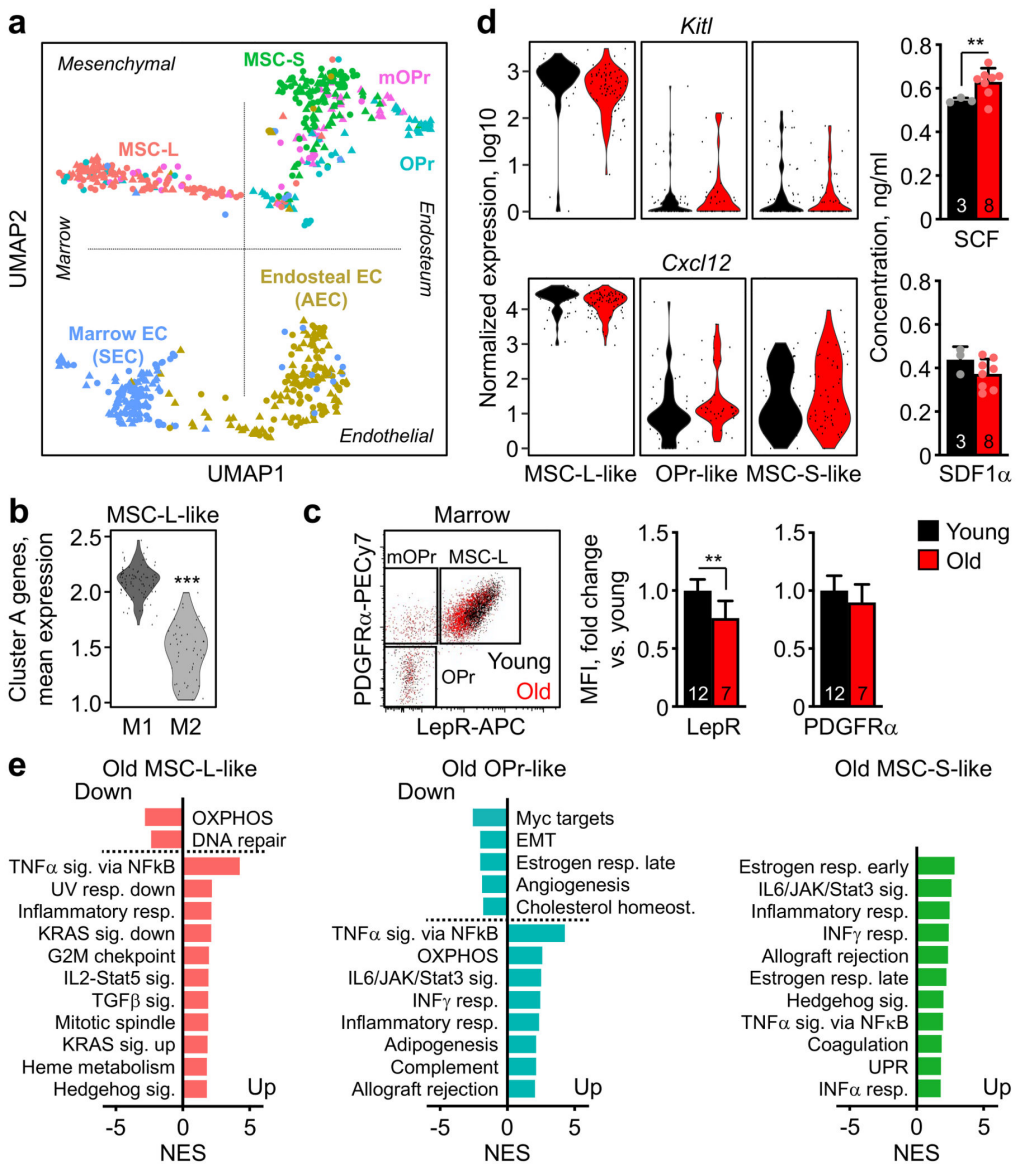
Molecular features of old mesenchymal populations. **a,** UMAP visualization of the entire plate-based scRNAseq dataset of niche populations of mesenchymal and endothelial populations shown in Fig. 2a. **b**, Global changes in MSC-L gene identity in clusters M2 vs. M1 (***padj ≤ 0.001). **c**, Representative flow cytometry staining (top) and quantification of LepR and PDGF-Ra levels (bottom) in young and old MSC-L. Results are from 6 independently analyzed groups of 1 or 2 young or old mice. **d**, HSC niche factors with violin plots representation of *Kitl* and *Cxcl12* expression in the indicated young and old mesenchymal populations (left) and SCF and SDF1a levels in young and old BM fluids (right). **e**, GSEA results for Hallmark biological processes significantly affected in old MSC-L-like, OPr-like and MSC-S-like groups (FDR < 0.05). Data are means ± S.D. except for (b) and (d); **p ≤ 0.01.

**Figure 2:**
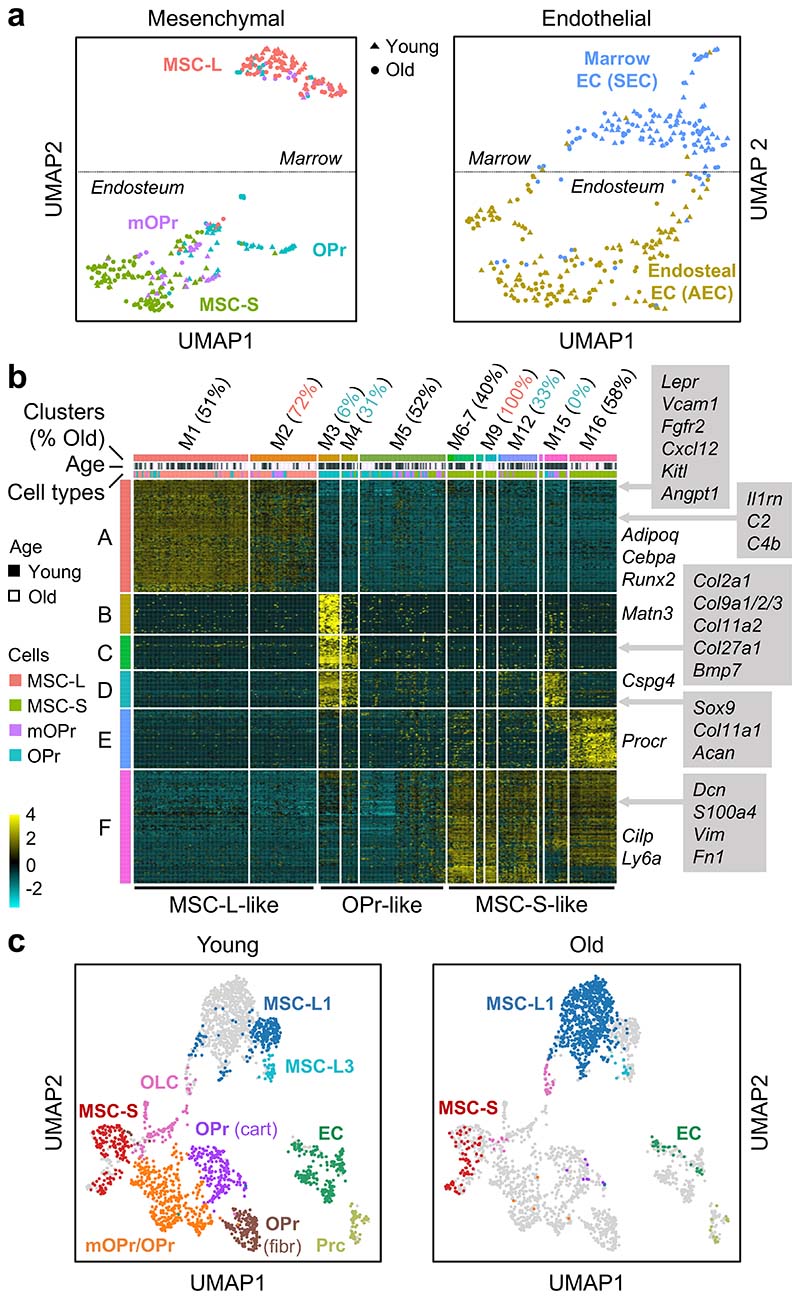
Molecular characterization of the old BM niche. **a**, UMAP visualization of plate-based scRNAseq analyses of mesenchymal (left) and endothelial (right) populations isolated from young (n = 3) and old (n = 4) mice. **b**, ICGS output of young and old mesenchymal populations with 16 clusters of cells (M1 to M16, horizontal) defined according to the expressing pattern of the 6 clusters of genes (A to F, vertical). Examples included in gene clusters A to F are shown. **c**, UMAP visualization of droplet-based scRNAseq analyses of endosteal and central marrow stroma fractions isolated from young (n = 2) and old (n = 1) mice. OLC, osteolineage cell; OPr(cart), cartilagenic osteoprogenitor; OPr(fibr), fibrogenic osteoprogenitor; Prc, pericyte.

To understand the molecular changes driving the old niche remodeling, we performed plate-based single cell RNA sequencing (scRNAseq) analyses on defined mesenchymal and endothelial populations isolated from both endosteum and central marrow of young and old mice. Uniform manifold approximation and projection (UMAP) representation confirmed preservation of the overall niche structure with age (Extended Data Fig. 2a; Supplementary Information SI.2a). Further visualization of mesenchymal populations indicates that endosteal MSC-S/mOPr/OPr exist along a continuum of cell states that is transcriptionally distinct from marrow MSC-L and revealed contamination of old endosteal populations by marrow MSC-L (Fig. 2a). Iterative clustering and guide-gene selection (ICGS) analyses identified 16 unique cell clusters, with M1-2 associated with MSC-L identity, M3-5 with OPr identity, and M6-16 with MSC-S identity (Fig. 2b). While most clusters had similar contribution from young and old cells, two consisted overwhelmingly of old cells (M2 and M9) while others had an under-representation of old cells (M3, M4, M12 and M15). Strikingly, M3/M4/M12/M15 all expressed osteoblastic and chondrogenic differentiation genes (Extended Data Table 1), supporting a loss of bone-forming cells with age. In particular, M3/M4 expressed high levels of collagens and other extracellular matrix genes, indicating that OPr-like cells are a more mature osteogenic population (Fig. 2b; Extended Data Table 1). In contrast, M9 was exclusively composed of old cells with downregulated bone formation pathways, while M2 was dominated by old cells with reduced expression of MSC-L identity genes and high expression of adipogenic marker genes (Fig. 2b; Extended Data Fig. 2b, Table 1). In fact, a large fraction of isolated old endosteal mOPr (45%) and OPr (58%) displayed M1 or M2 cluster identity gene expression, suggesting they were MSC-L that were sorted as OLC due to reduced LepR surface expression with age (Extended Data Fig. 2c). Categorization of scRNAseq data into MSC-L-like, OPr-like and MSC-S-like identity groups showed no changes in the expression of HSC maintenance factors *Kitl* and *Cxcl12* between young and old cells, with numerically expanded MSC-L likely contributing to elevated SCF levels in old BM fluids (Extended Data Fig. 2d). In contrast, gene set enrichment analysis (GSEA) of differentially expressed genes (DEG) between young and old cells within each identity group highlighted activation of inflammatory signaling pathways in all old mesenchymal populations (Extended Data Fig. 2e, Table S2). These molecular analyses demonstrate that the age-related loss of endosteal populations and expansion of marrow MSC-L are more pronounced than quantified by flow cytometry due to surface marker infidelity in old MSC-L. They also uncover a loss of osteoblastic/chondrogenic commitment and chronic activation of inflammatory signaling that permeates the mesenchymal hierarchy, providing a molecular basis for the functional deterioration of old endosteal populations and associated bone loss.

**Extended Data Figure 3:**
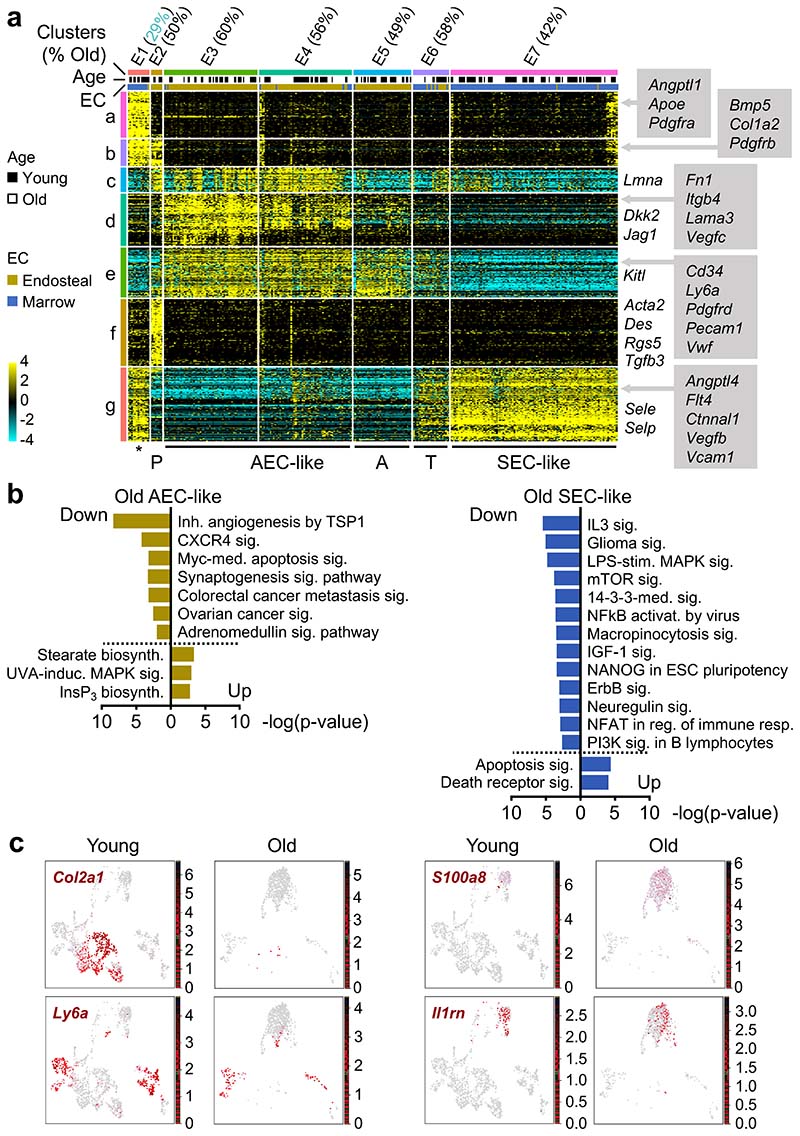
Molecular features of old endothelial populations and further characteristics of the old niche. **a**, ICGS output of young and old endothelial populations from the plate-based scRNAseq dataset shown in Fig. 2a with 7 clusters of cells (E1 to E7, horizontal) defined according to the expressing pattern of the 7 clusters of genes (a to g, vertical). Examples included in gene clusters a to g are shown. Star, contaminating mesenchymal/endothelial doublets; P, pericytes; A, arterioles; T, transition vessels. **b**, Ingenuity Pathway Analysis (IPA) canonical pathways enriched in old AEC-like and SEC-like groups (Z-score > 1; p < 0.01). **c**, Characteristic expression patterns for the indicated genes in the droplet-based scRNAseq dataset of young and old endosteal and central marrow stromal fractions shown in Fig. 2c. Cells in the UMAP were colored according to the expression levels of the indicated genes. Color scheme is based on ln scale of normalized counts from 0 (gray) to the indicated maximum value in the scale (red).

UMAP visualization of endothelial populations clearly separated marrow ECs (mostly composed of SECs according to index sorting data) from endosteal ECs (dominated by AECs) regardless of the age of the populations (Fig. 2a). ICGS analyses identified 7 cell clusters, with E3-4 associated with AEC-like identity and E7 with SEC-like identity (Extended Data Fig. 3a, Table 1). Ingenuity Pathway Analysis (IPA) of DEGs between young and old populations revealed repression of extracellular matrix genes, including many collagens, and activation of biosynthetic pathways in old AEC-like cells (Extended Data Fig. 3b, Table 1). In contrast, old SEC-like cells showed a broad downregulation of signaling pathways, decreased expression of endocytosis genes, and activation of cell death pathways. These molecular alterations likely underpin the dysfunctional and likely fragilized nature of old SECs, and confirm an overall loss of vascular integrity with age.

Finally, we complemented these molecular analyses with 10X Genomics droplet-based scRNAseq of unfractionated CD45^-^/Ter-119^-^ endosteal and marrow stroma from young and old mice. UMAP representation combining both locations was indexed using a published dataset^13^ and annotated using projections of identity groups from our plate-based scRNAseq dataset for cluster identification (Supplementary Information SI.2a,b,c). This approach confirmed the severe degradation of the endosteal niche with reduction in MSC-S and disappearance of almost all of the OLC and OPr subsets normally found in the young niche (Fig. 2c; Extended Data Fig. 3c). It also clearly captured the damaged vasculature found in old mice with a loss of EC clusters and illustrated the expansion of an inflammatory MSC-L1 subset of marrow MSC-L (Fig. 2c; Extended Data Fig. 3c). Collectively, these results provide a molecular atlas of the old niche and complement our flow cytometric and functional assays in demonstrating the loss of endosteal niche populations, fragilization of the vasculature, and inflammatory remodeling of central marrow MSC-L.

## Niche-mediated inflammation in the old BM milieu

**Figure 3:**
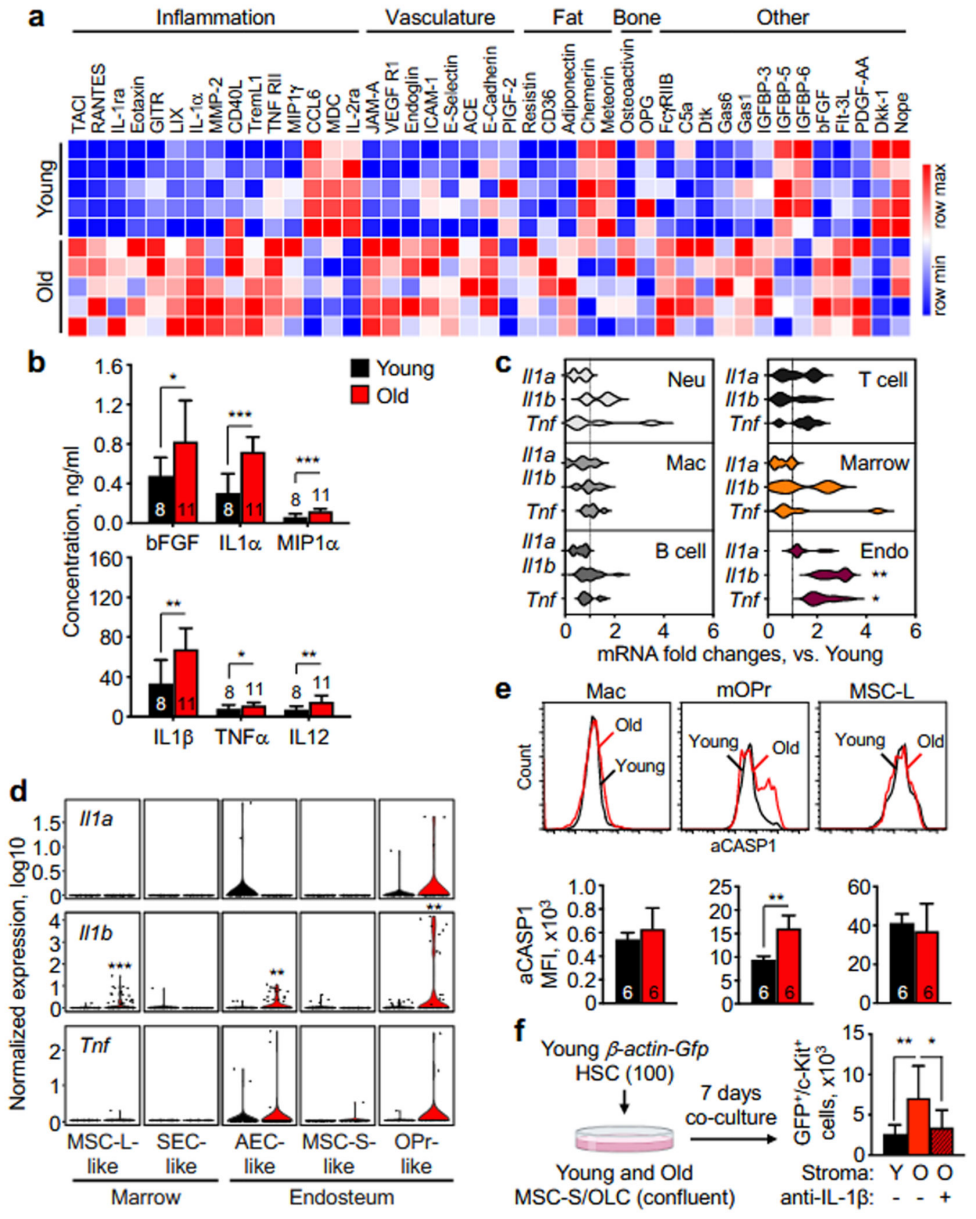
Inflammatory nature of the old BM niche. **a**, Differentially secreted cytokines in young and old BM fluid measured by 200-plex array and clustered based on broad biological functions (n = 5). **b**, Changes in pro-inflammatory cytokines in young and old BM fluids. Results are from 3 independent cohorts. **c**, qRT-PCR-based analyses of *Il1a*, *Il1b* and *Tnf* expression in old BM cells and stromal fractions (n = 6). Results are represented as violin plots and are expressed as fold change compared to their respective young counterpart. Neu, Mac-1^+^/Ly-6G^+^/Ly-6C^mid^ neutrophil; Mac, Mac-1^+^/Ly-6G^-^/Ly-6C^-^ macrophage; B cell, B220^+^/CD19^+^ B cell; T cell, CD4^+^/TCRβ^+^ T cell; Marrow, Ter119^-^/CD45^-^ central marrow fraction; Endo, Ter119^-^/CD45^-^ endosteal fraction. **d**, Violin plots representation of *Il1a*, *Il1b*, and *Il1rn* expression in the indicated young and old mesenchymal and endothelial like groups. Results are from the plate-based scRNAseq dataset; *padj ≤ 0.05, ** padj ≤ 0.01, *** padj ≤ 0.001. **e**, Caspase 1 activity (aCASP1) in the indicated young and old BM and stromal populations with representative FACS plots (top) and quantification (bottom). **f**, Experimental scheme for the indicated co-culture experiments showing the effects of young and old stroma on young HSC expansion as dependent on IL-1β. Data are means ± S.D.; *p ≤ 0.05, **p ≤ 0.01, ***p ≤ 0.001.

To determine how the old niche contributes to the establishment of an inflamed BM milieu, we used a 200-plex commercial array to screen cytokines present in BM fluids harvested from young and old mice (Fig. 3a). 81 analytes were detected at both ages, with 32 upregulated and 11 downregulated with age (Extended Data Table 3). The largest group represented inflammatory factors, some previously reported to increase with age such as RANTES^23^, Eotaxin (CCL11)^33^, and IL-1^24,25,34,35^. The second largest group comprised soluble ligands of endothelial cells like ICAM-1 and E-selectin, whose increased levels indicate vascular activation and inflammation^36^. Other categories included cytokines and chemokines involved in bone^37^ and fat^38^ production and function, which likely reflect the loss of endosteal OPr and the expansion of adipogenic marrow MSC-L^39^. Complementary analyses with cytokine bead-array measurements confirmed the pro-inflammatory state of the old BM milieu, with significantly elevated levels of IL-1α/β, MIP1α, and TNFα (Fig. 3b). These results reveal significant changes in secreted factors in the old BM milieu and complement previous studies reporting low-grade inflammation in the aged niche^20,23–25^.

**Extended Data Figure 4:**
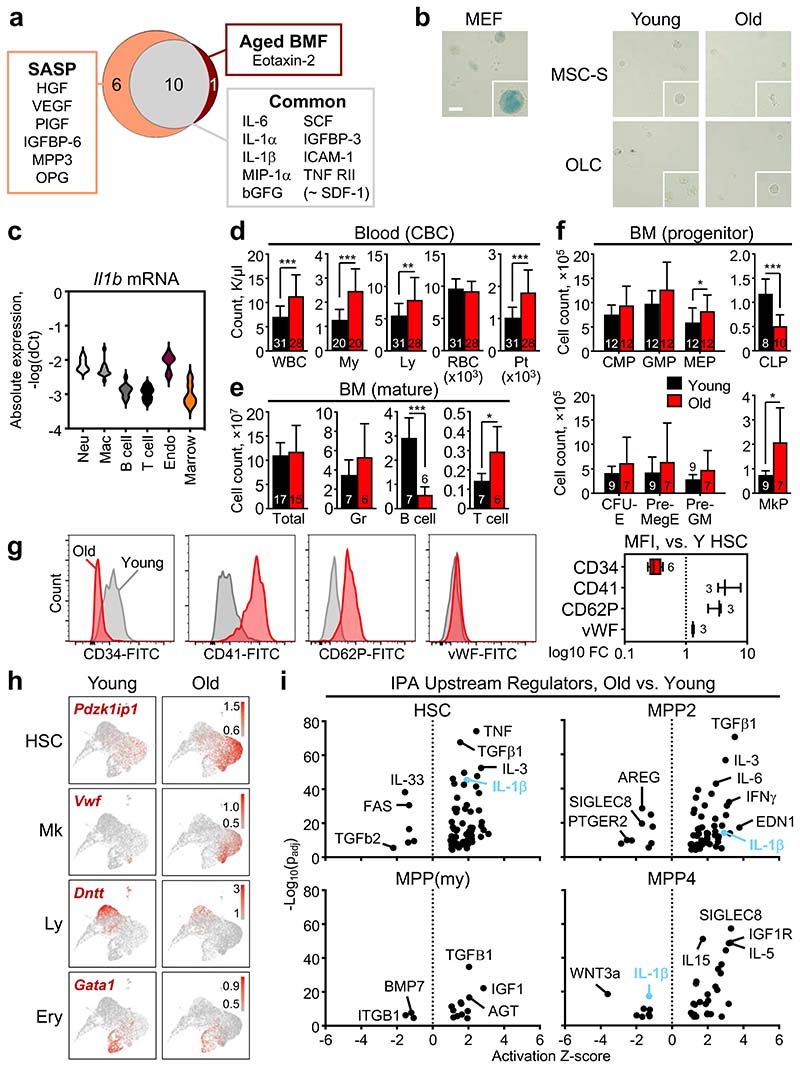
Age-related changes in blood and BM populations and altered lineage bias in old MPPs. **a,** Overlap between cytokines upregulated in old BM fluids and published SASP profile^40^. **b**, Representative SA-b-gal staining in control irradiated mouse embryonic fibroblasts (MEF) and isolated young and old MSC-S and OBC. Scale bar, 20 µm. **c**, Absolute expression of *Il1b* in different mature hematopoietic cell types and unfractionated (CD45^-^/Ter119^-^) endosteal and central marrow stroma from pooled young and old samples. Results are expressed as -log(dCt) relative to *gapdh*. **d**, Complete blood count (CBC) parameters in young and old mice. Results are from 7 independent cohorts. WBC, white blood cell; My, myeloid (neutrophil + basophil + eosinophil); Ly, lymphocyte; RBC, red blood cell; Pt, platelet. **e**, Cellularity and quantification of mature populations in young and old BM. Results are from 3 independent cohorts. Gr, Mac-1^+^/Gr-1^+^ granulocyte; B cell, B220^+^ B cell; T cell, CD3^+^ T cell. **f**, Quantification of progenitor populations in young and old BM. Results are from 3 independent cohorts. CMP, common myeloid progenitor; GMP, granulocyte-macrophage progenitor; MEP, megakaryocyte-erythrocyte progenitor; CLP, common lymphoid progenitor; CFU-E, erythroid colony-forming unit; PreGM, pre-granulocyte/macrophage; Pre-MegE, pre-megakaryocyte/erythrocyte; MkP, megakaryocyte progenitor. **g**, Representative staining and quantification of CD34, CD41, CD62P and vWF levels on young and old HSCs. Results are from 2 independent cohorts, with data represented as box and whiskers (min to max) and expressed as fold changes in mean fluorescence intensity (MFI) relative to young HSCs. **h**, Characteristic expression patterns of lineage determinant genes in the droplet-based scRNAseq young and old LK/LSK dataset shown in Fig. 4b. Cells in the UMAP were colored according to the expression levels of the indicated genes. Color scheme is based on ln scale of normalized counts from the indicated minimum (gray) to maximum (red) value in the scale. **i**, IPA Upstream Regulators analysis of young and old populations from the droplet-based scRNAseq young and old LK/LSK dataset shown in Fig. 4b filtered on cytokines and growth factors. Data are means ± S.D. except when indicated; *p ≤ 0.05, **p ≤ 0.01, ***p ≤ 0.001.

Although old BM fluid cytokines overlap with known senescence-associated secretory pathway (SASP) cytokines^40^, we did not find activation of senescence-related gene expression programs in any old mesenchymal populations (Extended Data Fig. 4a, Table 2). Isolated old endosteal MSC-S and OLC were also negative for senescence-associated β-galactosidase (SA-β-gal) staining (Extended Data Fig. 4b). To determine which old niche populations contribute to marrow inflammation, we then screened various types of BM mature blood cells including neutrophils, macrophages, B cells and CD4^+^ T cells (Supplementary Information SI.1c) as well as CD45^-^/Ter-119^-^ endosteal and central marrow stromal fractions for expression of *Il1a*, *Il1b* and *Tnf* by qRT-PCR. Strikingly, only the old endosteal stromal fraction showed increased *Il1b* and *Tnf* expression, with plate-based scRNAseq results confirming increased *Il1b* expression in endosteal OPr and AEC as well as marrow MSC-L (Fig. 3c,d; Extended Data Fig. 4c). While BM resident macrophages were previously identified as a source of IL-1β in old mice^24^, increased caspase 1 (CASP1) activity, the enzyme responsible for processing inactive pro-IL-1β into mature IL-1β, was only observed in old mOPr (Fig. 3e). To confirm that increased IL-1β production from old endosteal stromal directly affects HSC behavior, we co-cultured young HSCs on confluent MSC-S/OLC in the presence or absence of IL-1β blocking antibody. Strikingly, co-culture with old endosteal stroma increased HSC expansion, an effect that was abrogated upon IL-1β blockade (Fig. 3f), consistent with the known impact of IL-1 on HSC function^41^. These results demonstrate a key role of the old niche in driving marrow inflammation and identify the degraded endosteal OPr compartment as a relevant source of IL-1β with age.

## Inflammatory remodeling of the old blood system

**Figure 4:**
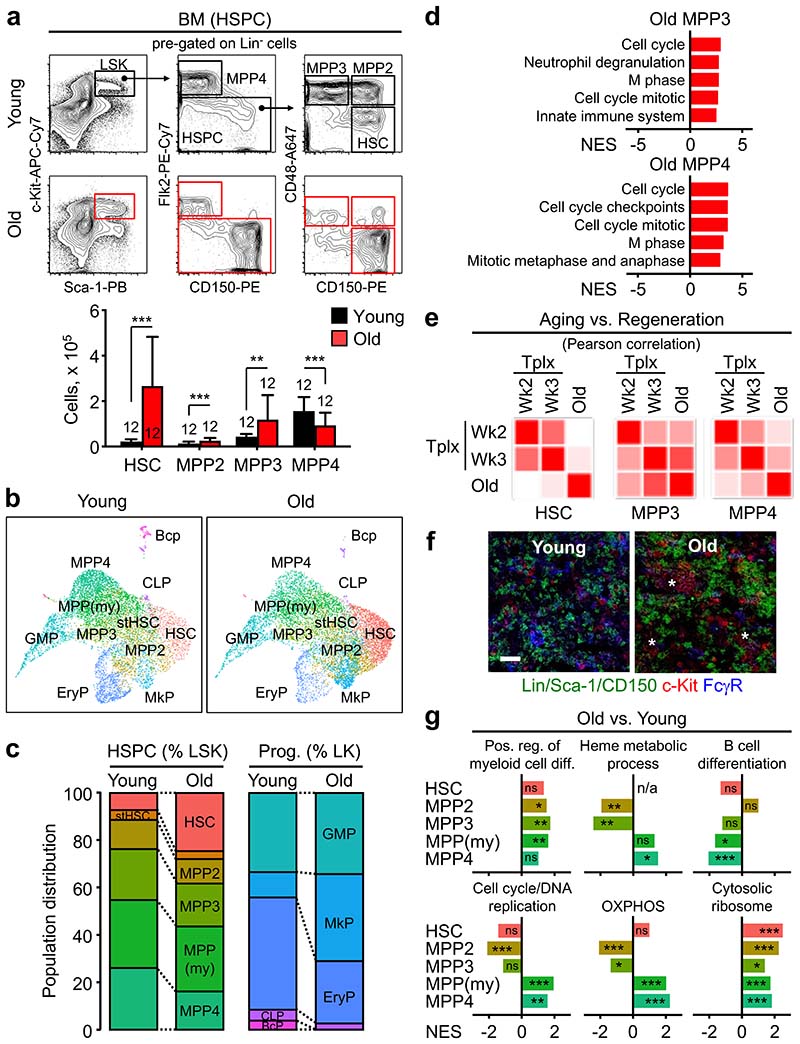
Activation of emergency myelopoiesis pathways in the old blood system. **a**, Representative flow cytometry plots (top) and quantification (bottom) of HSCs and MPP populations in young and old mice. Results are from 4 independent cohorts with data expressed are means ± S.D; **p ≤ 0.01, ***p ≤ 0.001. **b**, UMAP visualization of droplet-based scRNAseq analyses of Lin^-^/c-Kit^+^ (LK) and Lin^-^/Sca-1^+^/c-Kit^+^ (LSK) BM fractions isolated from young (n = 2) and old (n = 1) mice. stHSC, short-term HSC; MPP(my), myeloid-primed MPP; GMP, granulocyte/macrophage progenitor; EryP, erythroid progenitor; MkP, megakaryocyte progenitor; CLP, common lymphoid progenitor; BcP, B cell progenitor. **c**, Quantification of the HSPCs and progenitors (Prog.) identified by droplet-based scRNAseq analyses. Results are expressed as percent LSK and LK, respectively. **d**, GSEA results for Reactome pathway analyses of isolated young and old MPP3 and MPP4 analyzed by microarray (FDR < 0.05; n = 3 per population). **e**, Pearson correlation of Fluidigm gene expression data comparing old and regenerative HSCs, MPP3 and MPP4 (n = 4 per population). Regenerating populations were isolated 2 and 3 weeks following young HSC transplantation (Tplx)^6^. **f**, Representative image of GMP immunofluorescence staining in young and old BM. Stars indicate self-renewing GMP patches. Scale bar, 60 µm. **g**, GSEA results for Gene Ontology analyses of affected pathways in old populations identified by droplet-based scRNAseq analyses. n/a, non-available; ns, non-significant; *nominal p value ≤ 0.05, ** nominal p value ≤ 0.01, *** nominal p value ≤ 0.001.

To address the consequence of niche inflammation on the blood system, we first compared hematopoiesis in young and old mice (Fig. 4a; Extended Data Fig. 4d,e,f; Supplementary Information SI.1d). Old mice displayed elevated levels of white blood cells, relatively unchanged red blood cell (RBC) levels, and increased platelet (Pt) counts in peripheral blood that mirror the accumulation of mature Mks and Mk progenitors (MkP) in the BM^24,27^. In contrast, the numbers of BM resident myeloid cells and committed myeloid progenitors remained similar between young and old mice, while the number of common lymphoid progenitors (CLP) and B cells decreased with age. Hematopoietic aging has long been characterized by an expansion of the immature Lin^-^/c-Kit^+^/Sca-1^+^ (LSK) compartment, which contains HSCs and all MPP subsets^6^. This reflects a massive expansion of CD34^-^/CD41^+^ Mk-biased HSCs that also expressed other Mk-lineage markers like P-selectin and von Willebrand Factor (vWF)^26,42^, as well as increased production of myeloid-biased MPP2 and MPP3, and decreased numbers of lymphoid-biased MPP4^43^ (Fig. 4a and Extended Data Fig. 4f,g). To gain an unbiased view of immature HSPCs and their connection to downstream lineage-committed progenitors, we performed droplet-based 10X Genomics scRNAseq analyses of LSK and Lin^-^/c-Kit^+^ (LK) BM fractions from young and old mice. UMAP representation combining both fractions was indexed with marker gene expression for cluster identification^44,45^ (Supplementary Information SI.2d) and used to determine the main differentiation trajectories from HSCs, with MPP3 and MPP4 both contributing to a subset of myeloid-primed MPP(my) prior to GMP commitment, and MPP2 sitting atop the bifurcation between MkP and erythroid progenitors (EryP) (Fig. 4b; Extended Data Fig. 4h). This further quantified HSC and MkP expansion, and MPP4 contraction in old mice (Fig. 4c). Lastly, we harnessed the Upstream Regulators feature of the IPA software to identify the cytokines and growth factors predicted to drive alterations in the transcriptional state of HSCs and MPPs with age, which identifed a strong IL-1β signature in old HSCs and to a lesser extent in old MPP2 (Extended Data Fig. 4i), consistent with our findings in young mice that IL-1 acts on HSCs more potently than on MPPs^41^. These data confirm well-established changes in hematopoietic aging, demonstrate that inflammatory remodeling begins at the level of HSCs, and provide a molecular roadmap to further investigate the effect of niche inflammation on the aging blood system.

**Extended Data Figure 5:**
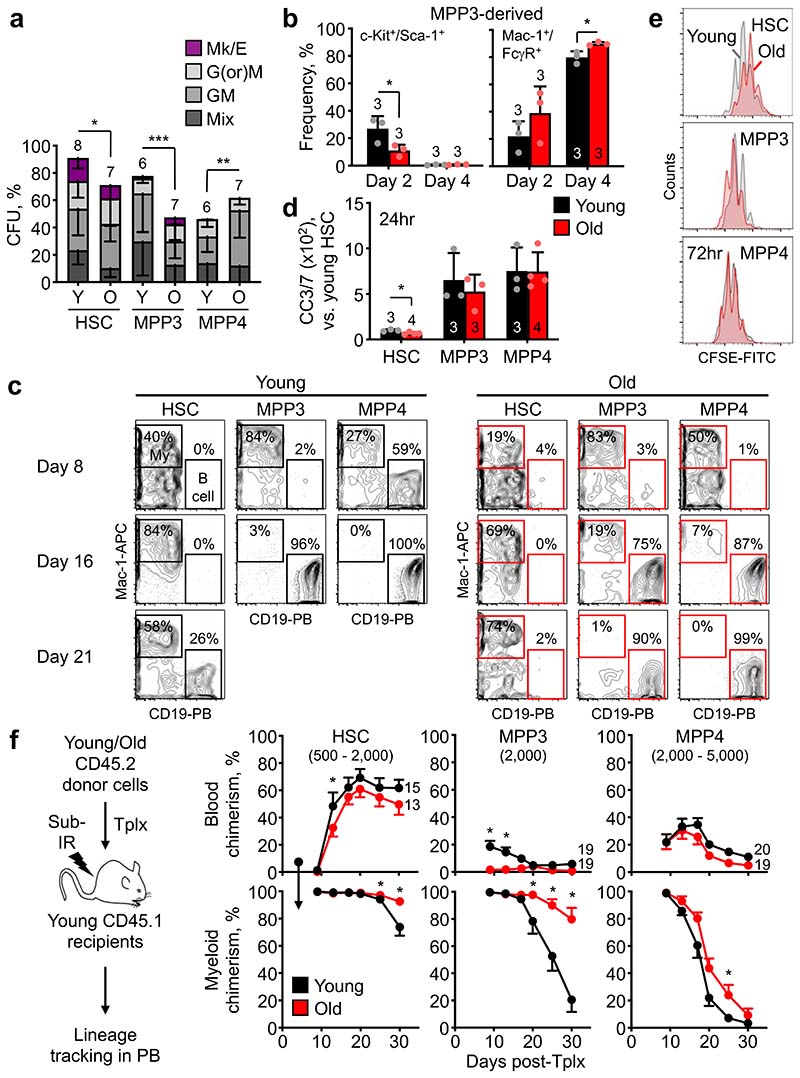
Altered functionality of old HSCs and MPPs. **a**, Colony formation in methylcellulose for young (Y) and old (O) HSCs, MPP3 and MPP4. Results are from 2 independent experiments. Mix: all lineages; GM: granulocyte/macrophage; G(or)M: granulocyte (or) macrophage; MegE: megakaryocyte/erythrocyte; CFU: colony-forming units. **b**, Myeloid differentiation in liquid culture for young and old MPP3 with quantification (right) of immature Sca-1^+^/c-Kit^+^ cells (left) and mature Mac-1^+^/FcgR^+^ macrophage (right). **c**, Representative flow cytometry staining of CD19^+^ lymphoid vs. Mac-1^+^ myeloid differentiation in OP9+IL7 culture conditions for young and old HSCs, MPP3 and MPP4. Results are representative of 3 independent experiments. **d**, Representative histograms of CFSE staining of cultured young and old HSCs, MPP3 and MPP4. Results are representative of 3 independent experiments. **e**, Cleaved caspase 3/7 (CC3/7) activity in cultured young and old HSCs, MPP3 and MPP4. **f**, Short-term lineage tracking following transplantations of young and old HSCs, MPP3 and MPP4 in sub-lethally irradiated recipients with experimental scheme (left) and quantification of overall blood donor chimerism (top graphs) and myeloid chimerism among donor cells (bottom graphs). Results are from 3 independent cohorts. Data are means ± S.D. except for (f) (± S.E.M.); *p ≤ 0.05, **p ≤ 0.01, ***p ≤ 0.001.

To determine the functional consequences of those changes, we next investigated the behavior of old MPP populations. *In vitro*, old MPP3 showed reduced clonogenic potential in methylcellulose and accelerated myeloid differentiation in liquid culture (Extended Data Fig. 5a,b). In contrast, old MPP4 had elevated myeloid colony-forming activity and, similarly to MPP3 and HSCs, delayed B cell differentiation in OP9/IL-7 co-cultures (Extended Data Fig. 5c). However, none of the old MPP populations exhibited delayed division kinetics associated with replication stress^46^ or dampened apoptotic responses^47^ characteristic of old HSCs (Extended Data Fig. 5d,e). Transplantation and short-term lineage-tracking confirmed the reduced reconstitution activity of old MPP3 and highlighted a strong myeloid bias from every old population including MPP4 (Extended Data Fig. 5f). These results confirm altered lineage differentiation potential with age, with enhanced myeloid commitment at the expense of lymphopoiesis that is reminiscent of emergency myelopoiesis programs^6,48^. They also indicate that old MPPs do not share key cell-intrinsic hallmarks of HSC aging.

To gain further insight into the molecular basis of this reprogramming, we first extended our published microarray analyses of the young hematopoietic hierarchy to include old HSPCs^6^. GSEA supported the unique molecular signature of old MPPs, with old MPP4 showing upregulation of cell cycle and DNA repair genes, and old MPP3 overexpression of genes associated with innate immunity and myeloid differentiation (Fig. 4d and Extended Data Table 4). Complementary Fluidigm-based qRT-PCR analyses comparing aging and regenerative signatures^6^ highlighted the correlation between old and 3-week post-transplantation MPP3 and MPP4, and to a lesser extent HSCs, further supporting a model whereby localized inflammation constitutively triggers myeloid regeneration programs in old HSPCs (Fig. 4e). Consistently, we also observed the presence of self-renewing GMP patches in the old BM cavity (Fig. 4f), which were never found in young mice absent a regenerative challenge^48^. Finally, GSEA of our scRNAseq results confirmed enhancement of myeloid/megakaryocyte and reduction of erythroid/lymphoid commitment at steady state (Fig. 4g). They also highlighted the differential metabolic/cell cycle activation of old MPP2 (down) vs. old MPP3/MPP4 (up) and the overall increase in ribosomal protein expression throughout the HSPC hierarchy. These results collectively illustrate profound alterations to hematopoiesis with age, with constitutive activation of myelopoiesis and megakaryopoiesis and suppression of lymphopoiesis and erythropoiesis. They also indicate steady-state engagement of emergency myelopoiesis pathways in old mice, with HSC activation, increased MPP3 production, myeloid-reprogramming of MPP4, and GMP cluster formation, consistent with chronic exposure to niche inflammation.

## Impaired hematopoietic recovery in old mice

**Figure 5:**
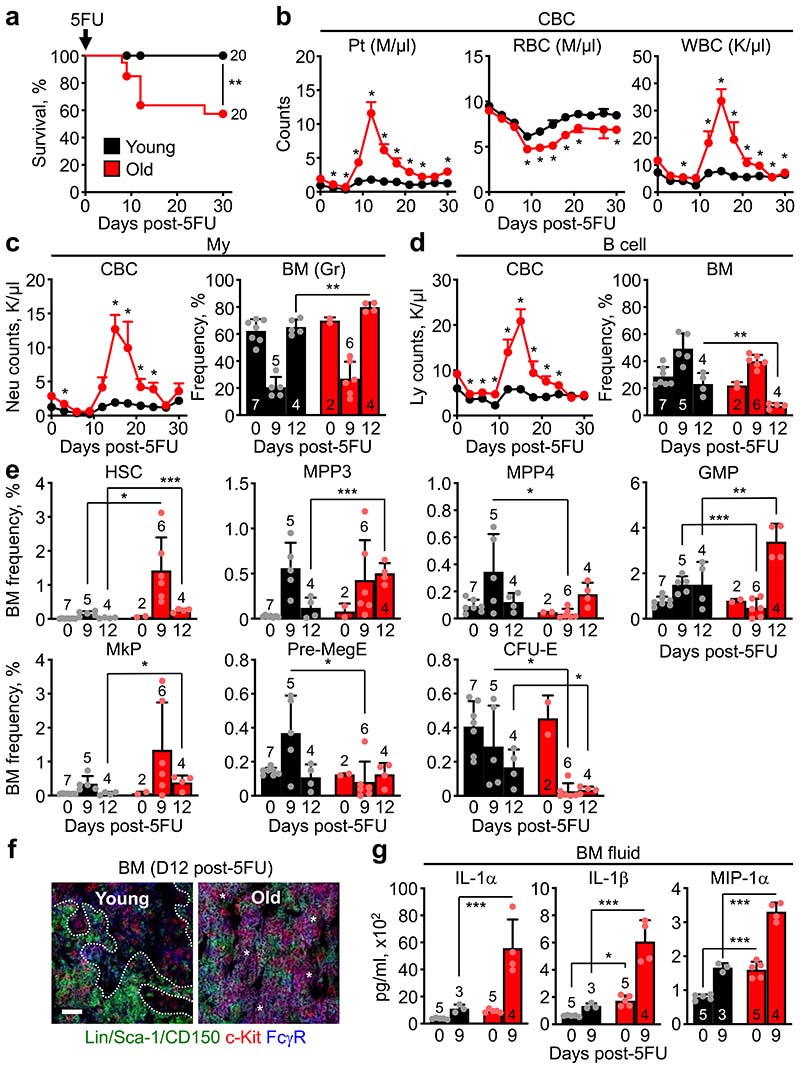
Impaired hematopoietic regeneration in old mice. **a-b**, Summary of (a) survival and (b) blood regeneration in young and old mice following one 5FU injection. Results are from 3 independent cohorts started with 20 individual young and old mice injected with 5FU per group. **c-d**, Regeneration of (c) myeloid and (d) B cell populations post-5FU treatment of young and old mice with quantification of changes in the blood (left) and BM (right). **e**, Regeneration of the indicated BM progenitor populations post-5FU treatment of young and old mice. **f**, Representative image of GMP immunofluorescence staining at 12 days (D12) post-5FU treatment of young and old mice. Dotted lines indicate differentiating GMP clusters and stars self-renewing GMP patches. Scale bar, 60 µm. **g**, Changes in IL-1α and IL-1β levels in BM fluids post-5FU treatment of young and old mice. Data are means ± S.D. except for CBC data shown in (b), (c) and (d) (± S.E.M.); *p ≤ 0.05, **p ≤ 0.01, ***p ≤ 0.001.

Impaired hematopoietic regeneration is a defining feature of aging that constrains BM donor eligibility and hinders clinical chemotherapeutic usage^4^. To understand how the old blood system responds to hematopoietic stress, we subjected young and old mice to 5-fluorouracil (5FU)-mediated myeloablation. Old mice were exquisitely sensitive to 5FU with ~ 50% mortality associated with massive thrombocytosis/thrombosis and anemia (Fig. 5a,b). Blood analyses revealed a severely altered regenerative response with age, with overproduction of mature myeloid cells, B cells and Pts and a persistent defect in erythropoiesis (Fig. 5b,c,d). BM analyses confirmed increased myeloid cell production, but showed decreased B cell numbers in the marrow, suggesting that emigration rather than increased production contributed to their elevated circulating levels in 5FU-treated old mice. In addition, we observed an amplified HSC response associated with increased and significantly delayed MPP and myeloid progenitor activation, delayed GMP cluster differentiation, and impaired erythroid progenitor (Pre-MegE and CFU-E) production in 5FU-treated old mice (Fig. 5e,f). Quantification of the local cytokine milieu indicated an exacerbation of niche inflammation in 5FU-treated old mice, with elevated IL-1α, IL-1β and MIP-1α levels in old BM fluids consistent with the role of these cytokines in promoting myelopoiesis and thrombopoiesis^41,49^ (Fig. 5g). These results suggest that by constitutively activating emergency myelopoiesis in old HSCs, age-related niche inflammation compromises acute hematopoietic regeneration and exacerbates myeloid and platelet biases.

## Chronic IL-1β exposure drives features of niche aging in young mice

**Figure 6:**
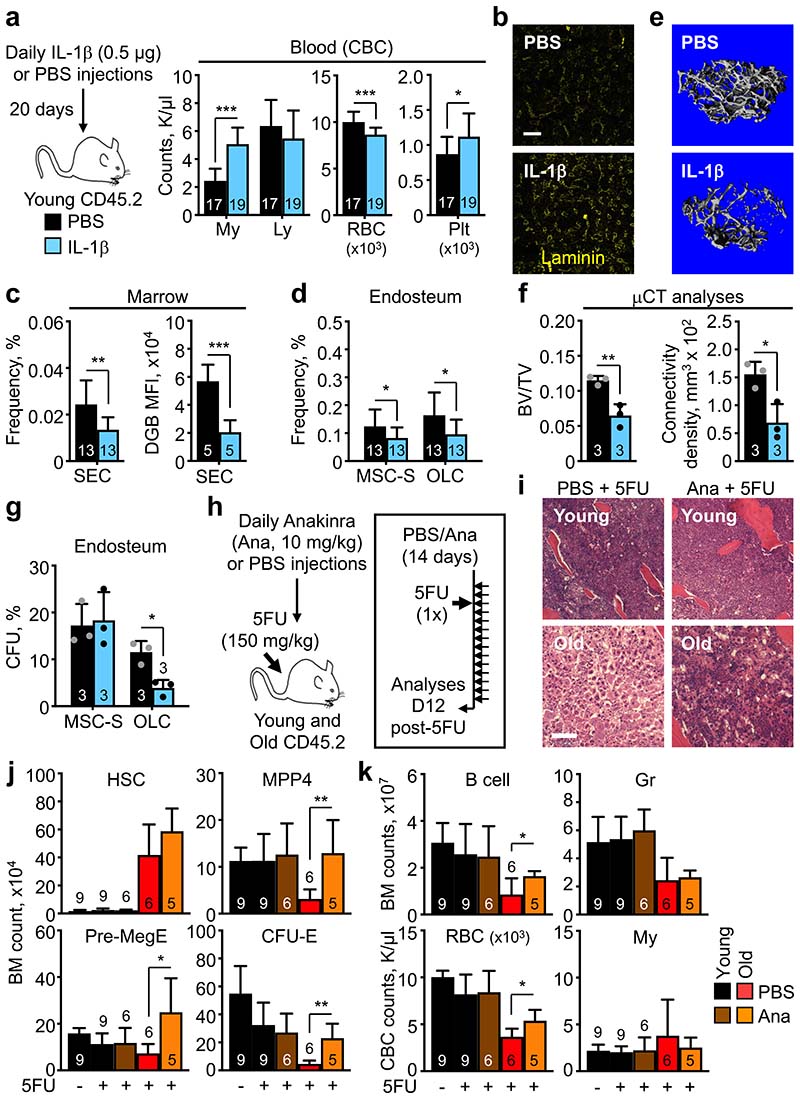
Modulating IL-1 levels in young and old mice affect aging parameters. **a-g**, Pro-aging effects of chronic IL-1β exposure in young mice with: (a) experimental scheme (left) and CBC values (right); (b) representative staining of the BM vasculature (scale bar, 100 µm); (c) changes in SEC frequency (left) and dragon green beads (DGB) retention (right); (d) changes in the indicated endosteal mesenchymal populations; (e) representative μCT images of trabecular bone; (f) quantification of μCT parameters; and (g) results of CFU-F assays. Results are from 4 independent cohorts of young and old mice injected daily with either PBS or IL-1β for 20 days. **h-k**, Pro-regenerative effects of acute IL-1 signaling blockade with Anakinra (Ana) in 5FU-treated old mice with: (h) experimental scheme; (i) representative H&E staining of sternum (scale bar, 100 µm); (j) quantification of the indicated BM HSPCs; and (k) quantification of the indicated BM mature populations and blood parameters. Results are from 3 independent cohorts started with 15 young and 11 old mice treated once with 5FU, injected daily with either PBS or Anakinra, and analyzed at day 12 post-5FU treatment. Data are means ± S.D.; *p ≤ 0.05, **p ≤ 0.01, ***p ≤ 0.001.

Chronic inflammation produced by daily injection in young mice of 0.5 µg IL-1β for 20 days mimics features of blood aging, including myeloid cell expansion, decreased lymphopoiesis, anemia and thrombocytosis (Fig. 6a) as well as impaired HSC engraftment^41^. Strikingly, we found that chronic IL-1β exposure also phenocopies several effects of aging on niche cells, with expansion of a dysmorphic and leaky sinusoidal network in the central marrow associated with SEC loss upon cell isolation, and decreased numbers of endosteal MSC-S and OLC (Fig. 6b,c,d). In addition, chronic IL-1β exposure induced morphological changes in bone and reduction in OLC potency that mimic normal aging as evidenced by µCT analyses and CFU-F assays (Fig. 6e,f,g). These results indicate that elevated IL-1 levels contribute to the functional deterioration of both the BM niche and the blood system.

## Improved hematopoietic regeneration upon acute IL-1 blockade in old mice

**Extended Data Figure 6:**
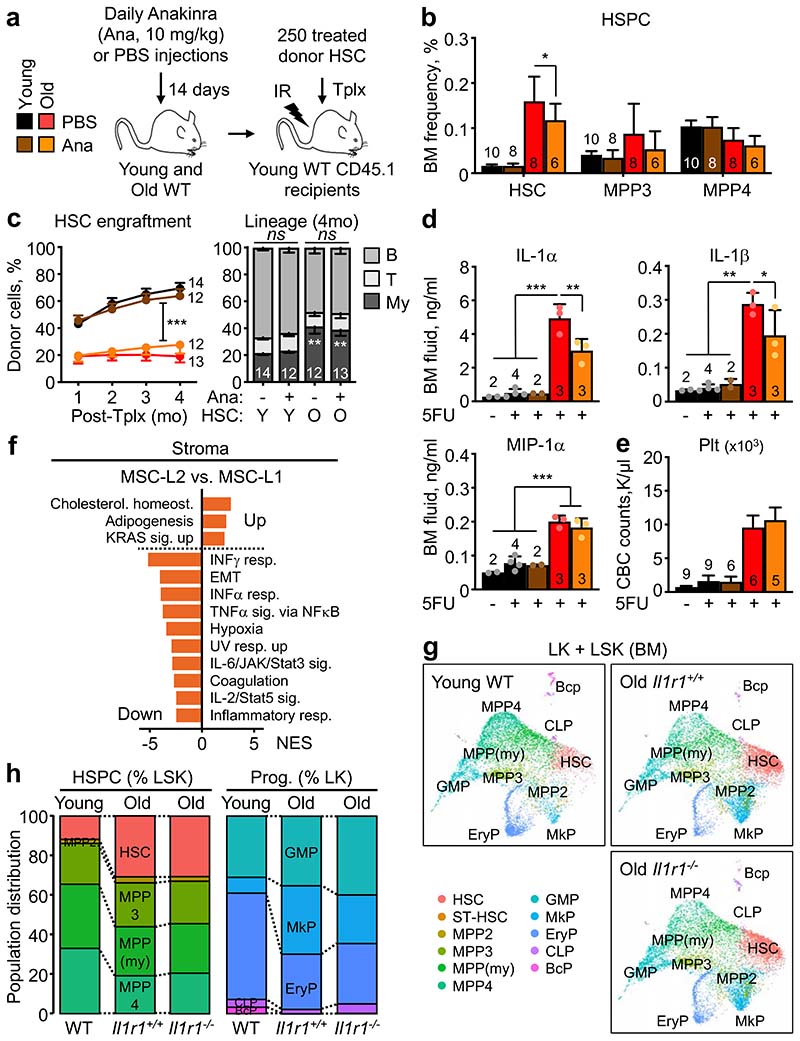
Improved aging features with IL-1 signaling blockade. **a-c**, Short-term blockade of IL-1 signaling upon Anakinra (Ana) treatment in young (Y) and old (O) mice with: (a) experimental scheme; (b) changes in HPSC frequency; and (c) engraftment over time (left) and lineage reconstitution (right) at 4 months (4 mo) post-transplantation (Tplx) of the indicated HSC populations. Results are from 3 independent cohorts of young and old mice injected with either PBS or Anakinra, with HSCs isolated from the pooled BM of mice from the same treatment group and transplanted into 3 to 5 recipients, each. **d-e**, Additional characterization of the effects of Anakinra blockade of IL-1 signaling during 5FU-mediated regeneration in young and old mice with: (d) changes in IL-1a, IL-1b and MIP1a levels in BM fluids; and (e) platelet (Plt) levels in peripheral blood. Results are from 3 independent cohorts started with 15 young and 11 old mice treated once with 5FU, injected daily with either PBS or Anakinra, and analyzed at day 12 post-5FU treatment. **f**, GSEA results for Hallmark biological processes significantly enriched in MSC-L2 vs. MSC-L1 groups (FDR < 0.05). Data are from the droplet-based scRNAseq analyses of endosteal and central marrow stromal fractions in young (n = 2) and old (n = 2) *Il1r1*^+/+^ wild type (WT) mice and old (n = 1) *Il1r1*^-/-^ mice shown in Fig. 7b **g-h**, Droplet-based scRNAseq analyses of Lin^-^/c-Kit^+^ (LK) and Lin^-^/Sca-1^+^/c-Kit^+^ (LSK) BM fractions isolated from young (n = 2) and old (n = 2) *Il1r1*^+/+^ wild type (WT) and old (n = 1) *Il1r1*^-/-^ mice with (g) UMAP visualization and (h) quantification of percent of HSPCs and progenitors. Data are means ± S.D. except for engraftment results shown in (c) (± S.E.M.); *p ≤ 0.05, **p ≤ 0.01, ***p ≤ 0.001.

To determine whether reducing IL-1 signaling could revert some aging features, we first administered the human IL-1 receptor antagonist Anakinra (Ana) for 14 days in young and old mice (Extended Data Fig. 6a). We used a short-term exposure paradigm to limit the risk of immune rejection, and consequently could not investigate an effect of Ana treatment on old BM niche cells that would have required longer treatment times to capture the slow turn-over time of stromal populations^29,50^. However, we found no effects of Ana treatment on steady-state hematopoiesis, with some changes in HSPC composition but no effects on old HSC functionality (Extended Data Fig. 6b,c; Supplementary Information SI.1e). Given the overproduction of IL-1α/β in old BM fluid following 5FU injection (Fig. 5g), we next investigated whether dampening IL-1 signaling to more youthful levels could improve hematopoietic regeneration. Strikingly, daily treatment with Ana starting 2 days before and continuing for 12 days after 5FU injection dampened IL-1α/β levels and significantly improved regeneration in old mice, with no discernable effect on the regenerative response of young mice (Fig. 6h,i and Extended Data Fig. 6d). In fact, we observed a recovery in B cell and RBC production in Ana-treated 5FU-injected old mice, which was directly accompanied by increased MPP4 and Pre-MegE/CFU-E numbers (Fig. 6j,k). IL-1β is well known to be required for effective myeloid recovery upon 5FU treatment, with 5FU-treated *Il1r1*^-/-^ mice having impaired hematopoietic regeneration^41^. In contrast, Ana treatment did not impair myeloid recovery in either young or old 5FU-treated mice, nor did it ameliorate thrombocytosis in old mice (Fig. 6k and Extended Data Fig. 6e), suggesting that it only provides short-term benefits accelerating acute regeneration when given at the height of IL-1 production in the BM niche. Collectively, these results demonstrate that short-term IL-1 blockade partially improves hematopoietic regeneration in old mice, with alleviation of anemia and recovery of B lymphopoiesis.

## Delayed niche aging and mitigated blood aging upon long-term IL-1 inhibition

**Extended Data Figure 7:**
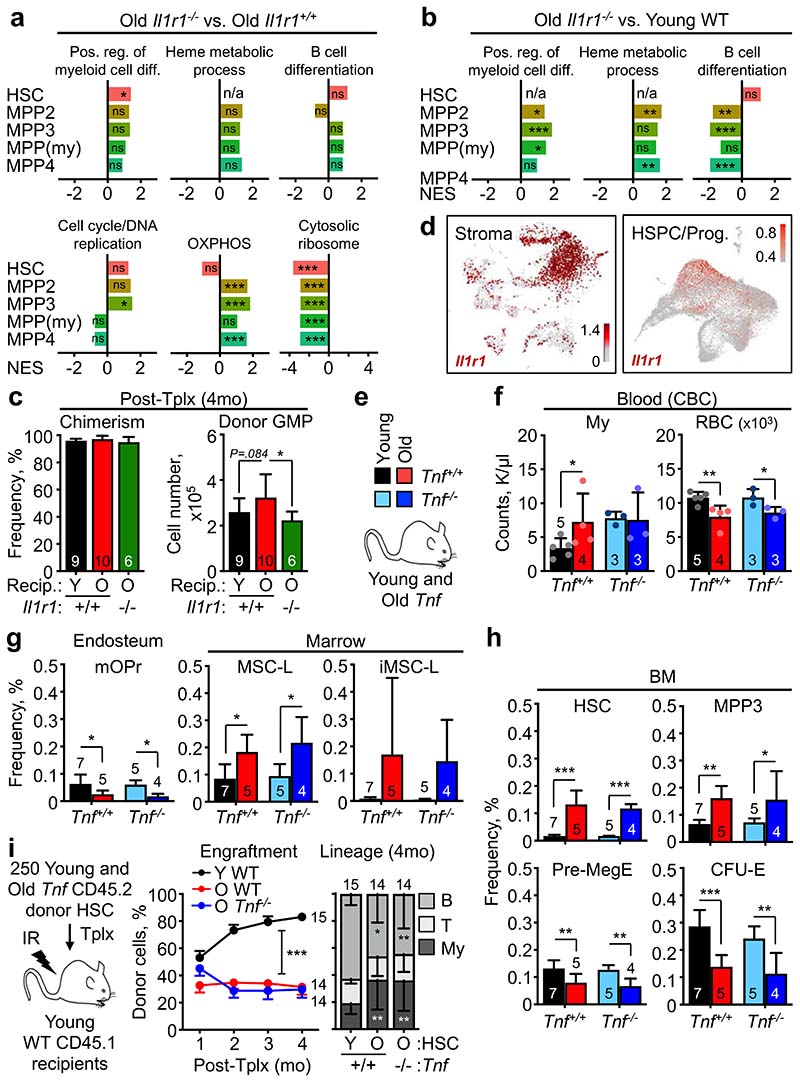
Key role of IL-1 in the aging of both BM niche and blood system. **a-b**, GSEA results in old *Il1r1*^-/-^ HSPC population for the Gene Ontology pathways affected in either old WT HSPCs (a) or young WT HSPCs (b) identified by droplet-based scRNAseq analyses. n/a, non-available; ns, non-significant; *nominal p value ≤ 0.05, ** nominal p value ≤ 0.01, *** nominal p value ≤ 0.001. **c**, Peripheral blood CD45.1^+^ donor chimerism (left) and number of donor-derived GMPs (right) in young or old WT and *Il1r1*^-/-^ CD45.2^+^ recipient mice at 4 months (mo) after lethal irradiation and transplantation (Tplx) with 2x10^6^ young WT CD45.1^+^ donor BM cells. **d**, *Il1r1* expression in the droplet-based scRNAseq of young and old WT stroma and LK/LSK datasets. Cells in the UMAP were colored according to the expression levels of the indicated genes. Color scheme is based on ln scale of normalized counts from the indicated minimum (gray) to maximum (red) value in the scale. **e-i**, Unchanged aging features in old *Tnf-/-* mice with: (e) color scheme; (f) blood parameters; (g) endosteal (left) and central marrow (right) mesenchymal population frequencies; (h) BM hematopoietic population frequencies; and (i) engraftment over time (left) and lineage reconstitution (right) at 4 mo post-Tplx of the indicated HSC populations. Results are from 3 independent cohorts of young and old WT and age-matched *Tnf*^-/-^ mice, with HSCs isolated from the pooled BM of mice of the same genotype and transplanted into 3 to 5 recipients, each. Data are means ± S.D. except for engraftment results shown in (i) (± S.E.M.); *p ≤ 0.05, **p ≤ 0.01, ***p ≤ 0.001.

**Figure 7:**
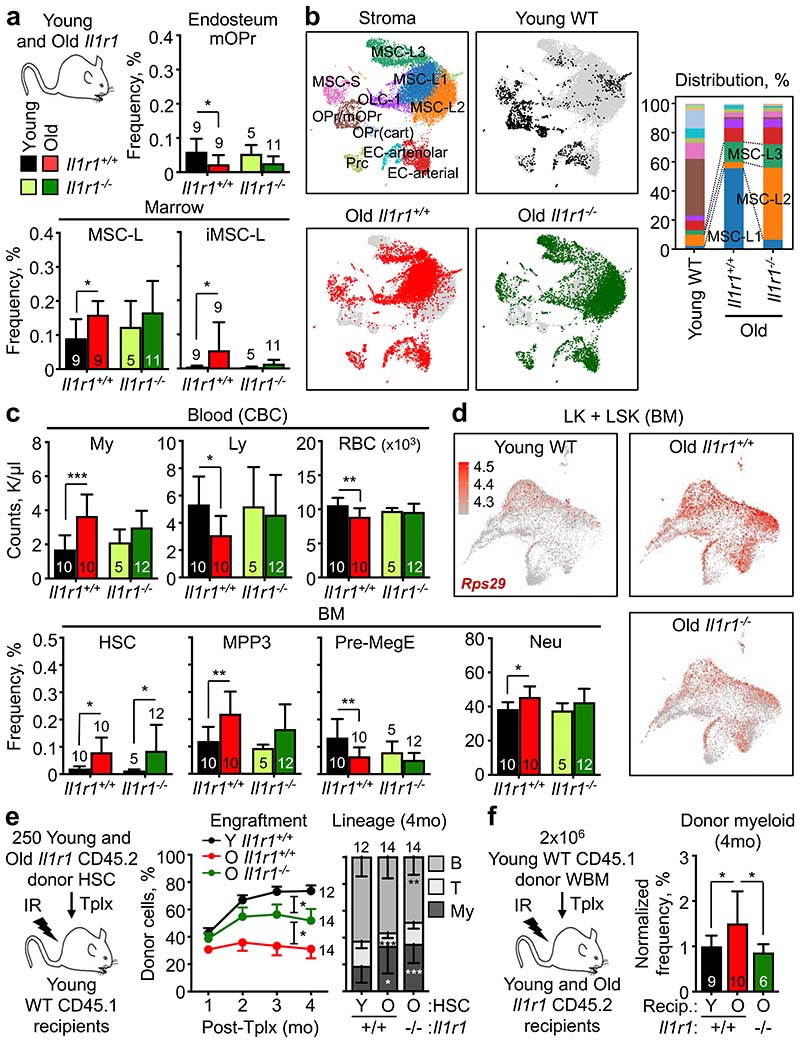
Blocking IL-1 signaling delays niche aging and improves old blood parameters. **a-b**, Improved stromal aging features in old *Il1r1*^-/-^ mice with: (a) color scheme (left) as well as endosteal (right) and central marrow (bottom) mesenchymal population frequencies; and (b) UMAP visualization of droplet-based scRNAseq analyses of endosteal and central marrow stromal fractions in young (n = 2) and old (n = 2) *Il1r1*^+/+^ wild type (WT) mice and old (n = 1) *Il1r1*^-/-^ mice with quantification of percent of the different mesenchymal and endothelial populations (right). **c-e**, Delayed blood aging and improved HSC function in old *Il1r1*^-/-^ mice with: (c) blood (top) and BM (bottom) parameters, (d) *Rps29* expression in the droplet-based scRNAseq of old WT/*Il1r1*^-/-^ LK/LSK dataset; and (e) engraftment over time (left) and lineage reconstitution (right) at 4 months (4 mo) post-transplantation (Tplx) of the indicated HSC populations. Results are from 3 independent cohorts of young and old WT and age-matched *Il1r1*^-/-^, with HSCs isolated from the pooled BM of mice of the same genotype and transplanted into 3 to 5 recipients, each. **f**, Experimental scheme and donor myeloid cell output in young and old WT and *Il1r1*^-/-^ recipients transplanted with young BM at 4 mo post-Tplx. Data are means ± S.D. except for engraftment results shown in (e) (± S.E.M.); *p ≤ 0.05, **p ≤ 0.01, ***p ≤ 0.001.

Finally, to test the necessity of IL-1 signaling in driving aging phenotypes, we analyzed young and old *Il1r1*^-/-^ mice together with age-matched *Il1r1*^+/+^ WT controls. Remarkably, old *Il1r1*^-/-^ mice displayed features of a more youthful niche, with limited loss of endosteal mOPr and expansion of marrow MSC-L occurring without iMSC-L induction (Fig. 7a). Molecular analyses with scRNAseq investigations of unfractionated CD45^-^/Ter-119^-^ endosteal and marrow stromal fractions confirmed these observations and highlighted a complete rebalancing from the inflammatory MSC-L1 subset to a more youthful MSC-L2 subset with decreased inflammatory signaling and normalized metabolism (Fig. 7b; Extended Data Fig. 6f). Old *Il1r1*^-/-^ mice also showed reduced features of blood aging both by immunophenotyping and droplet-based scRNAseq analyses of LK and LSK populations. In particular, none of the characteristic myeloid cell expansion, B cell loss, and anemia were observed in the blood of old *Il1r1*^-/-^ mice, and despite persistence of an expanded HSC compartment, we found an attenuation of MPP3 expansion with diminished myeloid cell production and limited loss of erythrocyte progenitors in old *Il1r1*^-/-^ BM (Fig. 7c). A more youthful rebalancing of MkP and EryP and a trend towards maintenance of the MPP4 compartment was confirmed at the molecular level (Extended Data Fig. 6g,h), although with only a modest reversion in GSEA lineage commitment pathways affected by aging in old *Il1r1*^-/-^ HSPCs compared to either old or young WT HSPCs (Extended Data Fig. 7a,b). However, old *Il1r1*^-/-^ HSPCs showed full recovery of OXPHOS activity and dampened ribosomal protein expression that was restored to almost youthful levels as shown for *Rps29* expression (Fig. 7d). In fact, old *Il1r1*^-/-^ HSCs exhibited increased fitness in transplantation assays, with significantly improved overall chimerism (Fig. 7e), which we speculate could be due to a rebalancing in proteostasis regulation^51^. To confirm the role of stromal IL-1 signaling in driving enhanced myelopoiesis in old mice, we also transplanted young BM cells into young or old WT and *Il1r1*^-/-^ recipient mice. Remarkably, the increased donor myeloid output observed in old WT recipients was rescued to youthful levels in old *Il1r1*^-/-^ recipients (Fig. 7f; Extended Data Fig. 7c). Taken together, these results demonstrate that life-long blockade of IL-1 signaling delays stroma aging and ameliorates specific aspects of blood aging by improving circulating levels of RBC and B cells and dampening myeloid cell production. They also indicate that increased IL-1β production by the degraded endosteal niche (mainly mOPr) acts in trans in damaging marrow MSC-L and affecting blood production by old HSCs, a conclusion supported by *Il1r1* expression pattern that was found strongly expressed by stromal MSC-L and BM MPP4, and much less expressed by other niche cells and HSPC populations (Extended Data Fig. 7d). The specificity of IL-1 in driving age-related niche inflammation was also substantiated by the totally unchanged aging features observed in old *Tnf*^-/-^ mice (Extended Data Fig. 7e,f,g,h,i). Collectively, these results offer a proof-of-concept for blocking specific niche-mediated chronic inflammatory signals as a strategy for improving blood production and hematopoietic regeneration with age.

## Discussion

**Extended Data Figure 8:**
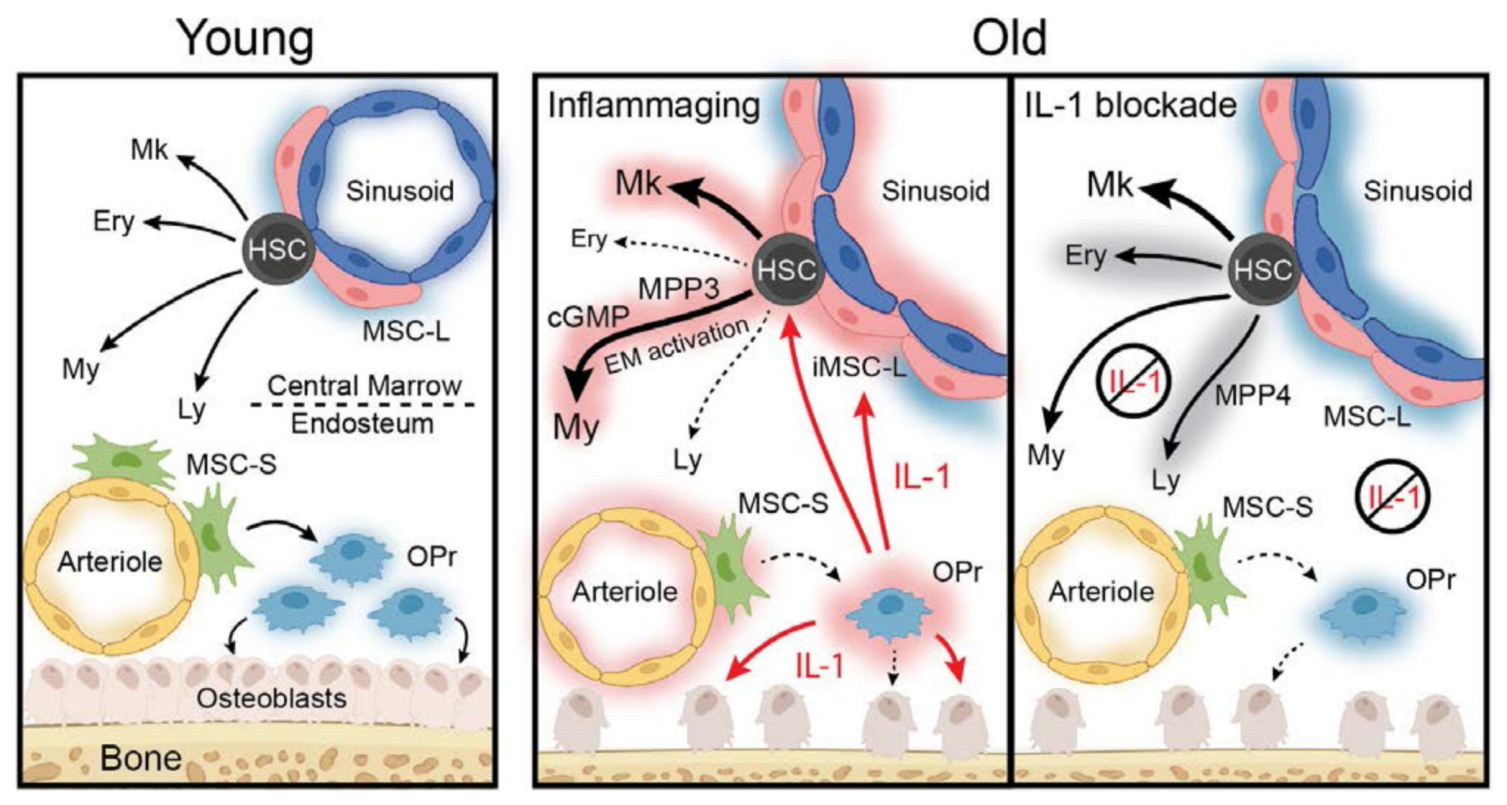
Schematic of the crosstalk between the BM niche and hematopoietic system during physiological aging. In youth, HSCs reside primarily in the central marrow where they are maintained by peri-sinusoidal MSC-L and produce a balanced output of all mature cell lineages (Mk, megakaryocytes; Ery, erythrocytes, My, myeloid cells; Ly, lymphoid cell). Abundant peri-arteriolar MSC-S at the endosteum efficiently produce OPr cells that support osteoblast development, ECM deposition and bone formation. With age, numerical loss and functional decline of MSC-S and OPr leads to bone thinning, with the remaining OPr constitutively producing IL-1. Chronic IL-1, in turn, reinforces niche degradation at the endosteum and contributes to dysfunction of the sinusoidal vasculature. Chronic IL-1 also acts in trans on central marrow MSC-L and HSPCs, driving the appearance of an inflammatory iMSC-L subset and steady-state engagement of emergency myelopoiesis (EM) programs with GMP cluster (cGMP) formation. Strikingly, acute IL-1 blockade with Anakinra enables more youthful blood production during 5FU-mediated regeneration, and life-long removal of IL-1 signaling in *Il1r1*^-/-^ mice maintains MSC-L in a more youthful cell state associated with improved blood production and HSC function.

Chronic inflammation is a hallmark of organismal aging, but its consequences for tissue function often remain unclear^10^. Here, we demonstrate that BM aging is defined by remodeling of the niche with increased production of pro-inflammatory cytokines by dysfunctional stromal cells and activation of inflammatory response programs in both hematopoietic and niche cells (Extended Data Fig. 8). Such chronic, low-grade “inflammaging” directly contributes to the loss of endosteal mesenchymal populations, impaired osteogenesis, and disorganization of sinusoidal blood vessels with increased vascular leakiness. These changes, alongside an expansion of inflammatory perisinusoidal MSC-L subset, creates a self-reinforcing cycle of damage that drives lineage biases and regenerative defects from the aged blood system. In particular, localized inflammation drives the constitutive activation of emergency myelopoiesis pathways from old HSCs and MPPs, reinforcing myeloid cell production at the expense of lymphoid and erythroid commitment. This in turn blunts regenerative responses that rely on acute activation of these pathways, leading to exacerbated phenotypes in stress conditions. Our results provide a novel understanding of blood aging based on crosstalk between the inflamed niche and inflamed hematopoietic system that degrades blood production both at steady state and during regeneration.

Our flow cytometry investigations coupled with scRNAseq analyses provide one of the most comprehensive descriptions of the aged BM niche to date. They add to previously published studies by illustrating the intrinsic fragility of old SECs that form the extended and dysmorphic sinusoidal network characteristic of old age, and by highlighting a previously unappreciated surface marker infidelity in the expanded old MSC-L populations that is a central feature of the inflammatory BM niche. High-definition imaging approaches have been used previously to investigate the vasculature in the old niche and identify a specific decrease in endomucin^bright^ transitional zone (type H) vessels at the endosteum that alter bone formation through impaired Notch signaling^17^. Our flow cytometry approach does not allow separation of those endothelial subsets, with *Emcn*-expressing type H vessels being captured in our broad marrow SEC-like group and their specific decrease with age overshadowed by the general loss of old SEC observed with digestion procedures for single cell preparation. These seemingly disparate results demonstrate the importance of using complementary investigation methods to understand different aspects of dysregulated endothelial cell biology with age. A reduction of endosteal niches and expansion of neurovascular central marrow niches promoting megakaryopoiesis through increased production of pro-inflammatory cytokines, including IL-6 and IL-1β, has also been previously described^20^. In addition, a role for IL-1β produced by aged macrophages has been implicated in promoting megakaryopoiesis and platelet production^24^, and by myeloid cells in response to changes in the microbiome for altering HSC function and promoting myelopoiesis^25^. Here, we expanded these findings by showing that chronic IL-1β production by the damaged endosteal niche, especially the few remaining osteoprogenitors, acts in trans by inducing inflammatory remodeling of the expanded population of perisinusoidal MSC-L and contributing to many aspects of altered blood production with age, particularly the changes in megakaryocyte/erythroid progenitor distributions and HSC activation/commitment to emergency myelopoiesis. It is likely that the indirect effect of MSC-L inflammatory remodeling triggered by IL-1 will also contribute to amplify blood deregulations. However, we found that blocking TNFα, one potential mediator of this indirect effect^52^, does not prevent neither niche nor blood aging, further highlighting the central role of IL-1. In fact, blocking IL-1 signaling attenuates central marrow MSC-L niche inflammation and helps dampen HSC activation, recovering age-related differentiation biases and improving the regenerative potential of the old blood system upon acute inhibition with Anakinra.

Our findings add to the growing body of evidence for an essential role of microenvironmental inflammation in blood aging, and the importance of IL-1β as a targetable driver of niche and blood aging^4,5,20,24,25^. They establish IL-1 as a central pioneering inflammaging factor that damages the crosstalk between niche and blood systems, with inflammatory remodeling of the central marrow likely having deleterious consequences for niche innervation^19,20^ and vascular function^17,18^ with age. Consistently, elevated IL-1β levels and activation of inflammasomes have been demonstrated in human studies and correlated with increased age-related mortality^53^. Finally, they indicate a novel therapeutic application of IL-1 inhibitors to improve blood production in the elderly, especially when hematopoietic regeneration is needed following chemotherapy or other immunosuppressive treatments.

## Methods

### Mice

Young and old wild-type C57BL/6-CD45.2 (B6) and wild-type C57BL/6-CD45.1 (BoyJ) mice of both sexes were bred and aged in house either at UCSF or CUIMC. Some old B6 mice were also obtained from the National Institute on Aging (NIA) aged rodent colonies and from collaborators. β-actin–GFP C57BL/6-CD45.2 transgenic mice^6^, *Il1r1*^-/-^ C57BL/6-CD45.2 mice^41^ and *Tnf*^-/-^ C57BL/6-CD45.2 mice^52^ were previously described. At the time of analyses, young mice were 6-12 weeks of age, middle-aged mice were 13 months old, and old mice were all ≥ 18 months of age, most of them ~ 24 month of age with some reaching 31 months of age. Young BoyJ recipient mice for HSC and MPP primary and secondary transplantation assays were 8-12 weeks of age at time of irradiation. No specific randomization or blinding protocol was used with respect to the identity of experimental animals, and both male and female animals were used indiscriminately in all experiments. All mice were maintained in mouse facilities at UCSF or CUIMC in accordance with IACUC protocols approved at each institution.

### *In vivo* assays

For quantitative Dragon Green Bead (DGB; Bangs Laboratories, FSDG001) analyses, mice were injected retro-orbitally with 2.5 µl/g DGB solution under isoflurane anesthesia 10 min prior to euthanasia by CO2 asphyxiation and immediately perfused with 20 ml PBS by cardiac puncture before bone harvest. For 5-fluorouracil (5FU; Sigma-Aldrich) treatment, mice were injected intraperitoneally with 150 mg/kg 5FU or vehicle (PBS) and analyzed for blood and BM parameters. For chronic IL-1 treatment, mice were injected intraperitoneally with 0.5 µg IL-1β (Peprotech, 211-11B) in 100 µl PBS/0.2% BSA or vehicle (PBS/0.2% BSA) daily for 20 days and analyzed for blood and BM parameters. For Anakinra treatment, mice were injected intraperitoneally with 10 mg/kg Anakinra (Swedish Orphan Biovitrum AB, 666-58234-07) or vehicle (PBS) daily for 14 days, with or without 5FU injection on the third day, and analyzed for blood and BM parameters. For transplantation experiments, CD45.1 recipient mice were exposed to 9 Gy (sub-lethal) or 11 Gy (lethal) irradiation dose delivered in split doses 3 hours apart using either a 137Cs source (J. L. Shepherd) or an X-ray irradiator (MultiRad225, Precision X-Ray Irradiation), and purified HSCs and MPPs were delivered via retro-orbital injection. For transplantation into sub-lethally irradiated recipients, mice were injected with 2,000-5,000 purified CD45.2 HSCs or MPPs. For transplantation in lethally irradiated CD45.1 recipients, mice were injected with 250 CD45.2 HSCs delivered together with 300,000 Sca-1-depleted CD45.1 helper BM cells. For reverse transplantations in lethally irradiated CD45.2 young and old WT and *Il1r1*^-/-^ recipients, mice were injected with 2x10^6^ young WT CD45.1 BM cells. Recipient mice were administered polymyxin/neomycin-containing water for 4 weeks following the procedure to prevent opportunistic infection and analyzed for blood and BM parameters. Peripheral blood was collected under isoflurane anesthesia via retro-orbital bleeding and dispensed into EDTA-coated tubes (Becton Dickinson) for complete blood count (CBC) analyses using a Genesis (Oxford Science) hematology system. BM analyses were terminal analyses at the time of tissue harvest and BM cellularity was determined using a ViCell automated cell counter (Beckman-Coulter).

### Flow cytometry of hematopoietic cells

BM hematopoietic stem, progenitor and mature cell populations were analyzed and/or purified as previously described^6^. In brief, BM cells were obtained by crushing leg, arm, and pelvic bones (with sternum and spines for some experiments) in staining media composed of Hanks’ buffered saline solution (HBSS) containing 2% heat-inactivated FBS (Cellgro B003L52). Red blood cells were removed by lysis with ACK (150 mM NH4Cl/10 mM KHCO3) buffer, and single-cell suspensions of BM cells were purified on a Ficoll gradient (Histopaque 1119, Sigma-Aldrich). For HSC and progenitor isolation, BM cells were pre-enriched for c-Kit^+^ cells using c-Kit microbeads (Miltenyi Biotec, 130-091-224) and an AutoMACS cell separator (Miltenyi Biotec). Unfractionated or c-Kit-enriched BM cells were then incubated with purified rat anti-mouse lineage antibodies (CD3, BioLegend, 100202; CD4, eBioscience, 16-0041-82; CD5, BioLegend, 100602; CD8, BioLegend, 100702; CD11b, BioLegend, 101202; B220, BioLegend, 103202; Gr1, eBioscience, 14-5931-85; Ter119, BioLegend, 116202) followed by goat anti-rat-PE-Cy5 (Invitrogen, A10691) and subsequently blocked with purified rat IgG (Sigma-Aldrich). Cells were then stained with c-Kit-APC-Cy7 (BioLegend, 105826), Sca-1-PB (BioLegend, 108120) or Sca-1-BV421 (BioLegend, 108128), CD150-PE (BioLegend, 115904) or CD150-BV650 (BioLegend, 115931), CD48-A647 (BioLegend, 115904) or CD48-A700 (BioLegend, 103426), and Flk2-biotin (eBioscience, 13-1351-85) or Flk2-PE (eBioscience, 12-1351-82) followed by SA-PE-Cy7 (eBioscience, 25-4317-82) for HSC/MPP staining, or together with CD34-FITC (eBioscience, 11-0341-85) or CD34-biotin (BioLegend, 119304) and FcγR-PerCP-Cy5.5 (eBioscience, 46-0161-82) or FcγR-PE-Cy7 (BioLegend, 101317) followed by SA-BV605 (BioLegend, 405229) for combined HSC/MPP/myeloid progenitor staining. For quantification of HSC surface marker expression, CD41-FITC (eBioscience, 11-0411-82), CD62P-FITC (BD, 561923) and vWF-FITC (EMFRET, P150-1) were also used. For extended myeloerythroid progenitor staining, unfractionated BM cells were incubated with Lin/PE-Cy5 and then CD41-PE (BD, 558040) or CD41-BV510 (BioLegend, 133923), FcγR-PerCP-Cy5.5 or FcγR-PE-Cy7, CD150-APC (BioLegend, 115910) or CD150-BV650, and CD105-A488 (BioLegend, 120406) or CD105-BV605 (BD, 740425). For CLP staining, unfractionated BM cells were incubated with Lin/PE-Cy5, then cKit-APC-Cy7, Sca-1-PB, Flk2-biotin, and IL7R-PE (eBioscience, 12-1271-82) followed by SA-PE-Cy7. For mature cell analyses, depending on the experiments, BM cells were stained with Mac-1-PE-Cy7 (eBioscience, 25-0112-82), Gr-1-e450 (eBioscience, 57-5931-82), B220-APC-Cy7 (eBioscience, 47-0452-82), and CD3-FITC (BioLegend, 100306). For myeloid cell isolation for qRT-PCR analyses, BM cells were stained with CD3-PE-Cy5 (eBioscience, 15-0031-63), B220-PE-Cy5 (eBioscience, 15-0452-82), NK1.1-PE-Cy5 (BioLegend, 108716), CD11b-FITC (eBioscience, 11-0112-82), Ly-6C-APC-Cy7 (BioLegend, 128025) and Ly-6G-A700 (BioLegend, 127622). For lymphoid cell isolation for qRT-PCR analyses, BM cells were stained with B220-APC-Cy7, CD19-PE (eBioscience, 12-0193-82), TCRβ-PE-Cy7 (Invitrogen, 25-5961-80), CD4-PE-Cy5 (eBioscience, 15-0041-82), and CD8-A700 (Pharmigen, 557959). For *in vitro* differentiation assays, cultured cells were stained with c-Kit-APC-Cy7, Sca-1-PB, Mac-1-PE-Cy7 and FcγR-PE (eBioscience, 12-0161-83) for myeloid differentiation, or Mac-1-APC (eBioscience, 17-0112-82) and CD19-PB (BioLegend, 115523) for lymphoid differentiation on OP9 cells. For HSC chimerism analyses, HSCs were stained with Lin/PE-Cy5, c-Kit-APC-Cy7, Sca-1-BV421, CD150-BV650, CD48-A700, and Flk2-bio followed by SA-BV605, together with CD45.2-FITC (eBioscience, 11-0454-85) and CD45.1-PE (eBioscience, 25-0453-83). For peripheral blood chimerism analyses, cells were stained with Mac-1-PE-Cy7, Gr-1-e450, B220-APC-Cy7, CD3-APC (eBioscience, 17-0032-82), and Ter-119-PE-Cy5 (eBioscience, 15-5921-83) together with CD45.2-FITC and CD45.1-PE. Before isolation or analyses, stained cells were resuspended in staining media containing 1 µg/ml propidium iodide (PI) for dead cell exclusion. HSC and progenitor cell isolations were performed on a Becton Dickinson (BD) FACS Aria II (UCSF) or FACS Aria II SORP (CUIMC) using double sorting for purity. Mature cell isolations were performed on a FACS Aria II SORP using single sorting on purity mode. Cell analyses were performed either on the same FACS ARIA, or on a BD LSR II (UCSF), BD Celesta (CUIMC), Bio-Rad ZE5 (CUIMC) or Novacyte Quanteon (CUIMC) cell analyzer. For all experiments, HSCs were identified as Lin^-^/Sca-1^+^/c-Kit^+^/Flk2^-^/CD48^-^/CD150^+^ BM cells, MPP3 as Lin^-^/Sca-1^+^/c-Kit^+^/Flk2^-^/CD48^+^/CD150^+^ BM cells, MPP4 as Lin^-^/Sca-1^+^/c-Kit^+^/Flk2^+^ BM cells, and, except when indicated, granulocytes (Gr) cells as Mac-1^+^/Gr-1^+^ BM cells, B cells as B220^+^ BM cells and T cells as CD3^+^ BM cells.

### Flow cytometry of niche cells

Endosteal stromal cells were isolated as previously described^29^. In brief, leg, arm, and pelvic bones were gently crushed and washed with HBSS until white. Stromal cells were released by treatment with 3 mg/ml Type I Collagenase (Worthington) in 2 ml HBSS for 1 hr at 37°C with shaking at 110 rpm. Stromal cells were washed with HBSS + 4% FBS and filtered through 45 µm mesh into a polypropylene tube. Isolation of central marrow stromal cells was adapted from a published protocol^54^ using individual femurs with the femoral head cut off and kneecap removed to expose the growth plate. Intact BM plugs were carefully flushed with HBSS into polypropylene tubes by inserting 1 ml syringes with 22G needles into the growth plate. HBSS was discarded and stromal cells were released by digestion with 3 mg/ml Type I Collagenase (Worthington) in 1 ml HBSS for 10 min twice at 37°C with shaking at 110 rpm. After the second incubation the plugs were resuspended by pipetting and filtered through 45 µm mesh into a new tube. Both endosteal and central marrow stromal cells were stained with Ter119-PE-Cy5, CD45-APC-Cy7 (BD, 557659), CD31-PE (BD, 553373), Sca-1-A700 (eBioscience, 56-5981-82), CD105-BV786 (BD, 564746), CD51-BV421 (BD, 740062), PDGFRα-PE-Cy7 (eBioscience, 25-1401-82) and LepR-biotin (R&D Systems, BAF497) followed by SA-APC (eBioscience, 17-4317-82), and resuspended in HBSS with 4% FBS and PI. Both cell isolations and analyses were performed on a Becton Dickinson (BD) FACS Aria II (UCSF) or FACS Aria II SORP (CUIMC) using single sorting on purity mode. For all experiments, MSC-S were identified as Ter119^-^/CD45^-^/CD31^-^/Sca-1^+^/CD51^+^, OLC as Ter119^-^/CD45^-^/CD31^-^/Sca-1^-^/CD51^+^, mOPr as Ter119^-^/CD45^-^/CD31^-^/Sca-1^-^/CD51^+^/PDGFRα^+^/LepR-, OPr as Ter119^-^/CD45^-^/CD31^-^/Sca-1^-^/CD51^+^/PDGFRα^-^/LepR^-^ and AEC as Ter119^-^/CD45^-^/CD31^+^/Sca-1^hi^/CD105^lo^ endosteal cells, while MSC-L were identified as Ter119^-^/CD45^-^/CD31^-^/Sca-1-/CD51^+^/PDGFRα^+^/LepR^+^ and SEC as Ter119^-^/CD45^-^/CD31^+^/Sca-1^lo^/CD105^hi^ central marrow cells.

### *In vitro* assays

All cultures were performed at 37 °C in a 5% CO2 water jacket incubator (ThermoFisher). For methylcellulose colony assays, single cells were directly sorted into individual wells of a flat-bottom 96-well plate (Fisher Scientific, 353072) containing 100 µl methylcellulose (Stem Cell Technologies, M3231) supplemented with penicillin (50 U/ml)/streptomycin (50 μg/ml), 0.1 mM non-essential amino acids (Fisher Scientific, 11-140-050), 1 mM sodium pyruvate (Fisher Scientific, 11-360-070), 2 mM L-glutamine (Fisher Scientific 35-050-061), 50 µM 2-mercaptoethanol (Sigma, M7522) and the following cytokines (all from PeproTech): IL-3 (10 ng/ml), GM-CSF (10 ng/ml), SCF (25 ng/ml), IL-11 (25 ng/ml), Flt-3L (25 ng/ml), TPO (25 ng/ml) and EPO (4 U/ml). Colonies were visually scored after 7 days of culture. For myeloid differentiation assays, 1,000 cells were directly sorted into individual wells of a 96-well plate containing Iscove’s modified Dulbecco’s media (IMDM) medium (Invitrogen) supplemented with 5% FBS (StemCell Technology, 06200) and the same reagents and cytokine cocktail than the methylcellulose assays (full cytokine medium). Cells were analyzed by flow cytometry after different culture periods. For cleaved caspase 3/7 (CC3/7) assays, 400-600 cells were directly sorted in triplicate into 40 µl full cytokine medium in a 384-well plate and incubated for 24 hr before adding 40 µl of Caspase-Glo 3/7 (Promega) to each well. Plates were then shaken for 30 s at 500 rpm, incubated for 45 min at RT and read on a luminometer (Synergy2, BioTek) to obtain relative units (RU). OP9 stromal cells (ATCC, CRL-2749) were maintained in Minimum Essential Medium Eagle alpha modification (αMEM) medium (Invitrogen) supplemented with 10% FBS (Cellgro, B003L52), L-glutamine (2 mM), and penicillin (50 U/ml)/streptomycin (50 μg/ml), and split at 1:4 every 3-4 days as needed to avoid over-confluence. For lymphoid differentiation assays, 500 cells were directly sorted into individual wells of a 24-well plate containing 10,000 OP9 stromal cells in OptiMEM medium (Invitrogen) supplemented with 5% FBS (StemCell Technology), L-glutamine (2 mM), penicillin (50 U/ml)/streptomycin (50 μg/ml), 2-mercaptoethanol (50 μM), SCF (10 ng/ml), Flt3L (10 ng/ml), and IL-7 (5 ng/ml). Following sequential withdrawal of Flt3L and SCF upon 2-day intervals, cultures were maintained in IL-7 and analyzed by flow cytometry at various intervals. For CFSE analyses, 1,000-1,500 cells were directly sorted into individual wells of a 96-well plate containing staining media, washed with PBS, resuspended in 100 µl PBS containing 5 µM CFSE (Molecular Probes, C-1157), incubated for 5 minutes at RT, quenched with FBS, incubated for 1 min at RT, washed twice with staining media, resuspended in 200 µl full cytokine medium, incubated for 72 hr and analyzed by flow cytometry. Stromal cells were grown in stroma media consisting of αMEM supplemented with 10% FBS (CellGro), penicillin (50 U/ml)/streptomycin (50 μg/ml), and 2-mercaptoethanol (50 μM). For fibroblastic colony-forming unit (CFU-F) assays, endosteal MSC-S (15-300 cells) or OLC (170-300 cells) were sorted directly in 6-well plates containing 1.5 ml medium/well and cultured for 11 days with medium exchange every 2-3 days, before staining with Giemsa-Wright to score colonies of 25 or more cells. In contrast, marrow MSC-L (100-300 cells) were sorted directly into 6-well plates containing 1.5 ml of Dulbecco’s Modified Eagle Medium (Gibco) supplemented with 20% FBS (CellGro), penicillin (50 U/ml)/streptomycin (50 μg/ml), 2-mercaptoethanol (50 μM), and 10 µM ROCK inhibitor (TOCRIS, Y-27632) and cultured in hypoxic conditions (5% O2) for 8 days with medium exchange every 2-3 days, before staining with Giemsa-Wright to score colonies of 25 or more cells. For Von Kossa staining, 1,000 cells were sorted into stroma media that was supplemented after 2 days of culture with β-glycerol phosphate (3mM, Sigma G9891) and ascorbic acid-2-phosphate (50 μg/ml, Sigma A8950). Cell were grown until confluence with media changes every 2-3 days, then fixed in phosphate-buffered formalin (Fisher 23-245-684), stained with silver nitrate solution (1%, Electron Microscopy Sciences 26212-01) and rinsed with sodium thiosulfate (5%, Ricca Chemical R7866500), with PBS washes between steps, before imaging. For co-culture assays with β-actin-GFP MSC-S, 25 cells were directly sorted into 96-well plates containing 100 µl medium/well, then 1x10^5^ unfractionated BM cells in 100 µl medium were added and co-cultures were incubated for 10 days without medium replacement. On day 10, medium was aspirated, and adherent cells were washed twice with PBS, liberated with 50 µl 0.25% trypsin/EDTA (Thermo, 25200056), and counted by flow cytometry using 25 µl Absolute Counting Beads (Life Technologies, C36950). For co-culture assays with endosteal CD45^-^/Ter119^-^/CD31^-^/CD51^+^ cells (MSC-S and OLC gates combined), 1,000 cells were sorted directly into 50% DMEM/50% αMEM supplemented with 20% FBS, penicillin (50 U/ml)/streptomycin (50 μg/ml), 2-mercaptoethanol (50 μM), and 10 µM ROCK inhibitor and grown to confluence for 7-10 days with medium changed every 2-3 days. Upon reaching confluence, half the medium was replaced with IMDM supplemented with 5% FBS (Stem Cell Technologies), penicillin (50 U/ml)/streptomycin (50 μg/ml), 0.1 mM non-essential amino acids, 1 mM sodium pyruvate, 2 mM L-glutamine, 50 µM 2-mercaptoethanol, 50 ng/ml SCF, 50 ng/ml TPO, and either 20 μg/ml anti-mouse/rat IL-1β (Bio Cell, BE0246) or 20 μg/ml isotype control (Armenian hamster IgG, Bio Cell, BE0091), and 100 β*-actin-Gfp* HSCs were seeded on top of this stromal layer. Co-cultures were grown for 7 days with media replacement every 2 days before cell counting and flow cytometry analysis. For SA-β-Gal (Cell Signaling, 9860S) staining, MSC-S and OLC were sorted directly onto poly-L-lysine coated slides (Sigma, P0425) and stained according to manufacturer’s protocol. For assessing Caspase 1 activity, analysis was performed using the FAM-FLICA Caspase-1 (YVAD) Assay Kit (ImmunoChemistry Technologies, 97) according to manufacturer’s instructions.

### Bone analyses

Bones for hematoxylin/eosin (H&E) staining were processed by the Mouse Pathology Core Facility at UCSF or the Molecular Pathology Core Facility at CUIMC. Tibia for MicroCT analyses were fixed in 10% neutral buffered formalin for 24 hours, then held in 70% ethanol at 4ºC until further processing. CT scans were performed on a vivaCT40 MicroCT (Scanco; 55 kV x-ray energy, 10.0 µm voxels, 500 ms integration times) and bone densitometry analyses were performed as previously described^29^. Trabecular tissue volume (TV), mineralized bone volume (BV), and trabecular bone parameters were determined on 100 slices, starting 500 µm below the bottom edge of the growth plate using segmentation values of 0.5/2/350 which corresponds to 650 mg hydroxyapatite/cm^3^.

### Immunofluorescence staining

For whole mount imaging, mice were injected retro-orbitally under isoflurane anesthesia with 100 µl of CD31-A647 (BioLegend, 102416) and VE-Cadherin-A647 (BioLegend, 138006) antibody cocktail in PBS. Injected mice were euthanized 10 min later, perfused with 3 ml PBS and fixed by perfusion of 10 ml 4% paraformaldehyde (PFA) in PBS. Femurs were harvested, fixed in 4% PFA for 2 hr on ice, washed 3x with PBS for 10 min each, and then cleared by successive dehydration with sucrose (15% and then 30%) for either 1 hr or overnight at each step. Bones were then snap-frozen in a 100% ethanol/dry ice slurry and kept at -80 °C until sectioning. One side of the frozen femur was cryosectioned with a tungsten blade (Leica, CM3050 S) until the BM was exposed, then transferred to a 1.5mL Eppendorf tube, washed 3x with PBS at RT and blocked with 20% goat-serum in PBS/0.1% Tween-20 (PBST) overnight at 4°C. Bones were incubated with rabbit anti-mouse laminin (Sigma, L9393) in 10% goat-serum in PBST for 3 days at 4°C, then washed 3x with PBST and incubated with donkey goat anti-rabbit A594 (Thermo Fisher, A32740) for 2 days at 4°C. Finally, bones were washed 3x with PBST for 10 min each, mounted on a coverslip with silicone glue, always kept wet with PBS, and imaged on an SP8 inverted confocal microscope (Leica) with 20x objective. For BM section imaging, mouse femurs were embedded in OCT, snap-frozen in a 100% ethanol/dry ice slurry and kept at -80 °C until sectioning. Thin 7 μm section were obtained upon cryosection using the CryoJane tape transfer system (Leica, 39475205) and a tungsten blade. Sections were dried for 2-4 hr at room temperature and then frozen at -80 °C until stained. Before staining, sections were fixed with 100% acetone for 10 min at -20 °C, dried for 5 min at RT, blocked for 90 min with 10% goat-serum (Gibco) in PBS and washes 3x with PBS. For LepR staining, sections were first stained with LepR-biotin (R&D systems, AF497) and rabbit anti-mouse laminin, and then with SA-A555 (Thermo Fisher, S21381) and goat anti-rabbit A594 (Thermo Fisher, A32740) followed by blocking with rat IgG for 10 min at RT. Slides were then stained with A488-conjugated lineage markers Ter119 (BioLegend, 116215), Mac-1 (BioLegend, 101217), Gr-1 (BioLegend, 108417), B220 (BioLegend, 103225), CD41 (Thermo Fisher, 11-0411-82) and CD3 (BioLegend, 100210). For ALCAM staining, sections were first stained with anti-ALCAM-Bio (R&D systems, BAF1172) followed by SA-A555 blocked with rat IgG and then stained with A488-conjugated lineage markers. For laminin staining, sections were stained with rabbit anti-mouse laminin followed by goat anti-rabbit-A647 and, eventually, DGB fluorescence was detected in the A488 channel. For GMP staining, sections were first stained with rat anti-mouse c-Kit (BioLegend, 135102) followed with a goat anti-rat-Cy3 (Jackson ImmunoResearch, 112-165-167), and then with A488-conjugated lineage markers, Sca-1-A488 (BioLegend, 108116), CD150-A488 (BioLegend, 115916) and FcγR-A647 (BioLegend, 101314). After staining, all sections were counterstained with 1 μg/ml DAPI in PBS for 10 min at RT, mounted with Fluoromount G (Southern Biotech, 0100-01) and imaged on an SP5 upright confocal microscopes (Leica) with 20x objectives. For DGB staining of purified SECs, 2,000 cells were sorted directly onto poly-L-lysine coated slides (Sigma-Aldrich, P0425-72EA) and allowed to settle for 10 min before being fixed with 4% PFA for 10 min at RT, washed and counterstained with 1 μg/ml DAPI in PBS for 10 min at RT, mounted with VectaShield (Vector Laboratories, H-1200) and imaged on an A1 Ti Eclipse inverted microscope (Nikon) with 20x objectives. Images were processed using Volocity software (Perkin Elmer v.6.2) and analyzed with ImageJ. The find objects function in Volocity was used to quantify vascular volume based on Laminin staining.

### Cytokine profiling

For each individual mouse, BM fluid was flushed out from the four hind leg bones (two femurs and two tibiae) using the same 200 µl of HBSS/2% FBS in a 1 ml syringe with 26G needle. BM cells were sedimented by centrifugation at 300 g for 5 min and collected supernatants were purified by an additional centrifugation at 15,300 g for 10 min. BM fluids were stored at -80°C until use. For 200-plex cytokine array, BM fluid samples were submitted to Quantibody Testing Service (Raybiotech) and diluted 4x prior to analyses. For bead array analysis, 50 µl of 2x diluted sample was analyzed using Mouse 20-Plex panel (Thermo Fischer Scientific) using either a Magpix (UCSF) or Luminex 200 (CUIMC) analyzer according to manufacturer’s protocol. For ELISA measurements, 4x-diluted (SCF, SDF1a, TPO; Raybiotech) and 90x-diluted (TGF-β; R&D Systems) samples were prepared according to the manufacturer's instructions and analyzed on a Synergy 2 plate reader (Biotek).

### Plate-based scRNAseq

Samples were processed following modifications to the Smart-Seq2 protocol^55^ based in the mcSCRB-Seq protocol^56^. Single endosteal and central marrow stromal cells were directly sorted into individual wells of a 96-well PCR plate in 2.3 µl of lysis buffer containing 0.2% Triton X-100 (Sigma-Aldrich) and 1U of Superase-In RNase Inhibitor (Ambion). Of note, information regarding expression of surface markers was recorded for each cell when sorting. Cells were frozen immediately at -80C until further processing. After thawing on ice, 2 µl of an annealing mixture containing1 µM oligodT (IDT), 5 mM each dNTPs and a 1:6,000,000 dilution of ERCC RNA Spike-In Mix (Invitrogen) was added followed by incubation at 72 °C for 3 min. Then 5.7 µl of a Reverse Transcription mix containing 3.5 U/µl of Maxima H minus retrotranscriptase (ThermoFisher), 0.88 U/µl of Superase-In RNase Inhibitor, 1.75x Maxima RT Buffer, 3.5 µM TSO (Qiagen) and 13.15% PEG 8000 (Sigma-Aldrich) was added and the mixture was incubated at 42 °C for 90 min, followed by 70 °C for 15 min. cDNA was further amplified by adding 40 µl of a PCR mix containing 0.03 U/µl of Terra PCR direct polymerase (Takara Bio), 1.25x Terra PCR Direct Buffer, and 0.25 µM IS PCR primer (ID). PCR was as follows: 3 min at 98 °C for initial denaturation followed by 21 cycles of 15 s at 98 °C, 30 s at 65 °C, 4 min at 68 °C. Final elongation was performed for 10 min at 72 °C. Sequences of oligodT, TSO and IS PCR primers were as previously described^55^. Following preamplification, all samples were purified using Ampure XP beads (Beckman Coulter) at a ratio of 1:0.6 with a final elution in 25 µl of EB Buffer (Qiagen). The cDNA was then quantified using the Quant-iT PicoGreen dsDNA Assay Kit (Thermo Fisher). Size distributions were checked on high-sensitivity DNA chips (Agilent Bioanalyzer). Samples were used to construct Nextera XT libraries (Illumina) from 100 pg of preamplified cDNA. Libraries were purified and size selected (0.5x-0.7x) using Ampure XP beads. Then, libraries were quantified using KAPA qPCR quantification kit (KAPA Biosystems), pooled and sequenced in an Illumina HiSeq 4000 instrument. Reads were mapped to the *Mus musculus* genome (EMSEMBL GRCm38.p4 Release 81) and ERCC sequences using GSNAP (version 2015-09-29) with parameters: -A sam –B 5 –t 24 –n 1 –Q –N 1. HTseq-count^57^ was used to count reads mapped to each gene, with parameters: –s no. All cells with < 100,000 reads mapping to endogenous RNA and >20% reads mapping to mitochondrial genes were considered low quality and removed from downstream analyses. Overall, 661cells (67.38%) passed our quality controls distributed as follows: Young marrow EC (SEC), 70; Old marrow EC (SEC), 71; Young endosteal EC (AEC), 84; Old endosteal EC (AEC), 82; Young MSC-L, 53; Old MSC-L, 58; Young MSC-S, 59; Old MSC-S, 59; Young mOPr, 26; Old mOPr, 26; Young OPr, 44; Old OPr, 29. The mean of nuclear reads mapped per cell was 471,244. Data were normalized and highly variable genes were identified as previously described^58^, using a false discovery rate threshold equal to 0.1 for the chi-squared test. Only highly variable genes were considered to perform PCA analysis, using the *prcomp* function in R (version 3.6.3). UMAP was calculated using *umap* function (version 0.2.7) in R based on normalized expression value with 15 nearest neighbors. All genes were used for ICGS clustering (AltAnalyzer)^59^ of mesenchymal and endothelial populations. In both cases we used version (v2.0) and ‘stringent’ with cell cycle and all other parameters as default. For mesenchymal populations, 16 clusters of cells (denoted M1 to M16) and 6 clusters of genes (denoted A to F) were defined. For endothelial populations, 7 clusters of cells (denoted E1 to E7) and 6 clusters of genes (denoted a to g) were defined. For subsequent analysis, mesenchymal clusters of cells were pooled as follows: M1 and M2 for “MSC-L-like”; M3, M4 and M5 for “OPr-like”; M6 to M16 for “MSC-S-like”. Subsequent endothelial groups of cells were defined as follows: clusters E3 and E4 constituted “AEC-like”; cluster E7 “SEC-like”. For MSC-L gene identity comparison, the geometric mean of the expression of all genes contained in cluster A was calculated for each cell of clusters M1 and M2 and then clusters M1 and M2 were compared using the independent two-sided t test (p-value < 2.2e-16). Differential expression analysis of old and young cells within each group was performed using DESeq2^60^ version 1.26.0. For violin plots representation, normalized expression values of *Kitl*, *Cxcl12*, *Il1a, Il1b* and *Il1rn* were plotted in various groups for young and old cells using the *ggplot2* package (version 3.3.0) in R with scale parameter set to ‘width’. Pathway analysis was conducted using Gene Set Enrichment Analysis (GSEA) software v4.1.0. Gene symbols were mapped to MSigDB.v7.2.chip and overlaps with Hallmark (h.all.v7.2.symbols.gmt) gene sets were determined using the classic scoring scheme. For endothelial cell types, no FDR<0.05 significant enrichments were obtained using GSEA, and so Ingenuity Pathway Analyses software (Qiagen, December 2020) was eventually employed. For IPA of endothelial cells, log 2 fold change of expression and p-adjusted values for all genes with nominal p values under 0.05 were imported into the software and IPA Canonical Pathways were determined.

### Droplet-based scRNAseq for stroma analyses

For both endosteal and central marrow populations, 30,000-50,000 Ter119^-^/CD45^-^ stromal cells were sorted into 1.5 ml tubes containing 500 µl of filter-sterilized αMEM with 10% FBS and transferred to the Columbia Genome Center Single Cell Analysis Core for microfluidic cell processing, library preparation and sequencing. In brief, cells were re-counted, and viability was assessed using a Countess II FL Automated Cell Counter (Thermo), and samples were processed following manufacturer’s recommendations for Chromium Single Cell 3' Library & Gel Bead Kit v2 (10X Genomics). 17,500 cells were loaded for each sample and 1 sample was loaded per condition. Samples were sequenced in Illumina HiSeq4000 sequencer machine. We obtained an average of ~286 million reads per sample. The alignment was done using *Cellranger* (version 2.1.1). Top 2,300 barcodes were selected for samples corresponding to young and old central marrow and young endosteum. Top 2,858 barcodes were selected for the sample corresponding to old endosteum. The downstream analysis was done using *Scanpy* version 1.6.0 (Wolf et al., 2018) in *Python* version 3.7.1. For these samples, 757 doublets were estimated and removed using *Scrublet* package version 0.2.1^61^. Further quality control (QC) was performed based on 3 parameters: 1) cells with at least 200 and no more than 7,000 genes detected; 2) cells with less than 65,000 associated counts; 3) cells with less than 5.5% of UMI counts associated to mitochondrial genes. After QC, 8,735 cells were kept for subsequent analysis. In addition, only genes that have more than 1 UMI count in at least 5 cells were maintained in further analysis. Cells were then normalized to 10,000 UMIs per cell and logarithmically transformed. Highly variable genes (HVGs) were selected using “highly_variable_genes” method with “min_mean=0.0175, max_mean=3, min_disp=0.5”. Read depth, number of genes and number of mitochondrial counts were removed using the “regress_out” function in *Scanpy*. UMAP visualizations were obtained from 50 PCA components and 10 neighbors using *Scanpy*. Clusters were defined using *Louvain* clustering version 0.7.0. Clusters containing stromal cells were subset based on the expression of genes expressed mainly in either hematopoietic cells or stromal cells. Only clusters that contained cells that expressed stromal marker genes were kept for subsequent analysis. In total, 2378 cells were selected, distributed as follows: 188 cells came from the young central marrow sample, 726 cells from the old central marrow sample; 1320 cells came from the young endosteum sample and 144 cells from the old endosteum sample. Highly variable genes (HVGs) were then obtained for the selected cells and the effects of read depth, number of genes and number of mitochondrial counts were regressed, as indicated above. The UMAP visualization was obtained as before and again clusters were defined using Louvain clustering with “*resolution 0.3*”. Cell types were annotated using typical marker genes for the different populations.

### Droplet-based scRNAseq for hematopoietic analyses

For both LK and LSK populations, 30,000-50,000 cells were sorted into 1.5 ml tubes containing 500 µl of HBSS containing 2% FBS and transferred to the Columbia Genome Center Single Cell Analysis Core for microfluidic cell processing, library preparation and sequencing as described above for stromal analyses. Downstream analyses done using *Seurat* version 4.0.3 in *R* version 4.1.0. Quality control (QC) was performed excluding cells with less than 200 genes detected or more than 7.5% of UMI counts associated to mitochondrial genes. In addition, only genes that have more than 1 UMI count in at least 3 cells were maintained in further analysis. In total, 28535 cells were selected, distributed as follows: 8489 cells came from the young WT sample, 9688 cells from the old WT sample; 10358 cells came from the old *Il1r1*^-/-^ sample. Cells were then normalized to 10,000 UMIs per cell and logarithmically transformed. Highly variable genes (HVGs) were selected using the “FindVariableFeatures” method. Cell cycle variation was removed using the “CellCycleScoring” method followed by regressing out “S.Score” and “G2M.Score”. Young WT and Old WT samples were integrated using the “IntegrateData” method (Stuart et al., 2019). UMAP visualizations were obtained from 10 PCA components and clusters were defined at a resolution of 0.4 using K-nearest neighbor graph-based. Cell types were annotated using typical marker genes for the different hematopoietic populations. Differential gene expression was performed using “FindMarkers” method on non-cell cycle regressed expression data. Old *Il1r1*^-/-^ data was analyzed by mapping to the Young WT/Old WT reference dataset using the “MapQuery” method^62^.

### Quantitative RT-PCR analyses

10,000-50,000 cells per population were sorted directly into RPE buffer (Qiagen) and stored at -80°C until purification with the RNeasy Plus Mini Kit (Qiagen) according to manufacturer’s protocol. Following column purification, RNA was immediately reverse transcribed using SuperScriptIII kit with random hexamers (Invitrogen). qPCR runs were performed on a QuantStudio 7 Flex Real-Time PCR System (Applied Biosystems) using SYBR Green reagents (Applied Biosystems), the cDNA equivalent of 200 cells per reaction template, and triplicate measurement per biological repeat. Sequences for qRT–PCR primers were: *Il1a*, forward GCACCTTACACCTACCAGAGT and reverse TGCAGGTCATTTAACCAAGTGG (NM_010554); *Il1b*, forward GCAACTGTTCCTGAACTCAACT and reverse ATCTTTTGGGGTCCGTCAACT (NM_008361); *Tnf*, forward AGGGATGAGAAGTTCCCAAAT and reverse GCTTGTCACTCGAATTTTGAG (NM_013693); and *Gapdh*, forward GACTTCAACAGCAACTCCCAC and reverse TCCACCACCCTGTTGCTGTA (NM_008084). Cycle threshold values were normalized to *Gapdh*.

### Fluidigm analyses

Gene expression analyses using the Fluidigm 96.96 Dynamic Array IFC were performed as previously described^6^. Briefly, HSC, MPP3 and MPP4 (100 cells/well) were directly sorted per well of a 96-well plate containing 5 µl CellsDirect lysis buffer (Invitrogen, 11753-100), reverse-transcribed and pre-amplified for 18 cycles using SuperScript III Platinum Taq Mix (Invitrogen, 12574-026) with a custom-made set of 96 proprietary target-specific primers (Fluidigm). The resulting cDNA was analyzed on a Biomark system (Fluidigm) using EvaGreen SYBR dye (Bio-Rad, 172-5211). Data were collected with Biomark Data Collection Software (Fluidigm) and analyzed using Biomark qPCR software with a quality threshold of 0.65 and linear baseline correction. Melt curves and melting temperature values for each assay reaction were checked individually, and reactions with melt curves showing multiple peaks or poor quality were discarded. Results for 2- and 3-weeks post-transplantation data were recalculated with *Hprt* as housekeeping gene and genes not analyzed in one of three conditions were removed, leaving 64-68 genes excluding housekeeping genes (*Actb*, *Gapdh*, *Gusb* and *Hprt*) for further analyses and calculation of Pearson’s correlation coefficients. For each dataset, values for each gene were averaged, and fold change values were calculated against young non-transplanted control and converted to Log2 value. Similarity matrixes were visualized with *Morpheus* (Broad Institute).

### Microarray analyses

Microarray analyses of old HSCs, MPP3 and MPP4 were performed as previously described^6^. Three to five independent biological replicates were used for each population. Total RNA was isolated from 20,000 cells per population sorted directly into TRIzol-LS (Invitrogen) and purified using Arcturus PicoPure (Applied Biosystems) with RNase-free DNase (Qiagen). RNA was amplified, labeled, and fragmented using NuGEN Ovation Pico linear amplification kits (Nugen Technologies) and hybridized onto mouse Gene ST 1.0 arrays (Affymetrix). Gene expression microarray data were normalized using RMA followed by quantile normalization as implemented in the 2.15.1 R package (www.r-project.org) using a standard (lambda=1) exponential reference distribution. Significance Analysis of Microarrays (SAM) (Tusher et al., 2001) was then performed on young and old cells within a population to determine SAM delta scores. Pathway analysis was performed using GSEA version 4.1.0 on the top 1000 up and downregulated genes in young versus old for each population. Gene symbols were mapped to MSigDB.v7.2.chip and overlaps with Reactome (c2.cp.reactome.v7.2.symbols.gmt) gene sets were determined using the classic scoring scheme.

### Quantitation and Statistical Analysis

All experiments were repeated as indicated; n indicates the numbers of independent biological repeats. Data are expressed as mean ± standard deviation (S.D.) or standard error of the mean (S.E.M.) as indicated. Mice for treatment and transplantation were randomized, samples were alternated whenever possible, and no blinding protocol was used. No statistical method was used to predetermine sample size. Pairwise statistical significance was evaluated by two-tailed Student’s t-test. P-values (p) ≤ 0.05 were considered statistically significant. For violin plot analyses of scRNAseq data, p-values for each comparison were obtained by two-tailed Student’s t-test and adjusted for multiple comparisons using the Benjamini-Hochberg method. Adjusted p-values (padj) ≤ 0.05 were considered statistically significant. Figures were made with GraphPad Prism software.

## Extended Data

**Extended Data Table 1:** ICGS of young and old niche cells. Plate-based scRNAseq gene expression data of mesenchymal and endothelial cells were separated and subjected to unsupervised single-cell population identification using Iterative Clustering and Guide-gene Selection (ICGS). The relative expression of every guide gene in each cluster, as well as the relative expression of every guide gene in each pooled group of clusters corresponding to the indicated cell types, was calculated by averaging the relative expression of each gene across individual cells within a cluster or group.

**Extended Data Table 2:** DEGs and pathway analyses of young versus old niche cells. Plate-based scRNAseq gene expression data between young and old cells in pooled identity groups as defined by ICGS were analyzed for differentially expressed genes (DEG) using the DESeq2 package. Overlap with Hallmark gene sets was evaluated, and statistically significant (FDR<0.05) overlaps are indicated in bold for mesenchymal populations. Since no significant overlaps were observed for endothelial populations, Ingenuity Pathway Analysis software (Qiagen) was employed to determine IPA Canonical Pathways with absolute z-score ≥ 1 and -log_10_(p-value) ≥ 2 enriched in young and old AEC-like and SEC-like cells.

Under detection limit in both young and old		
4-1BB	I-TAC	Marapsin
6Ckine	IFNg	MCP-1
Activin A	IFNg R1	MCP-5
ADAMTS1	IGFBP-2	MCSF
ALK-1	IL-1 R4	MIP-1a
ANG-3	IL-10	MIP-1P
ANGPTL3	IL-12p40	MIP-3a
AR	IL-12p70	MMP-10
Artemin	IL-13	OX40 Ligand
B7-1	IL-15	P-Cadherin
BLC	IL-17	Pentraxin 3
BTC	IL-17B	Persephin
CCL28	IL-17B R	Prolactin
CD27	IL-17E	Prostasin
CD27L	IL-17F	RAGE
CD30	IL-1P	SCF
CD30L	IL-2	SDF-1a
Chordin	IL-20	sFRP-3
CT-1	IL-21	Shh-N
CTLA4	IL-22	SLAM
DAN	IL-23	TARC
DLL4	IL-3	TECK
EDAR	IL-3 Rb	Testican 3
EGF	IL-33	TGFb1
Endocan	IL-4	TIM-1
Epigen	IL-5	TNFa
Epiregulin	IL-6	TPO
Fas	IL-7	TRANCE
Fas L	IL-7 Ra	Tryptase ε
G-CSF	IL-9	TSLP
Galectin-7	Kremen-1	TWEAK
GITR L	Leptin	VEGF
GM-CSF	Limitin	VEGF R2
Gremlin	Lungkine	VEGF R3
HAI-1	Lymphotactin	VEGF-B
HGF R	MadCAM-1	VEGF-D
Detected in all young but not old samples		
Eotaxin-2	IL-2 Ra	H60
TCA-3	Meteorin	TRAIL
TWEAK R	PlGF-2	
Detected in all old but not young samples		
Dtk	CD6	GITR
CD36	KC	
Above highest standards		
Clusterin	IGF-1	Periostin
Cystatin C	L-Selectin	TCK-1
Galectin-1	MBL-2	TNF RI
Galectin-3	P-selectin	VCAM-1
Not significantly changed		
BAFF R	HGF	Neprilysin
CD40	IL-28	NOV
CD48	Leptin R	OPN
CRP	Lipocalin-2	PF4
CXCL16	LOX-1	Pro-MMP-9
Decorin	MFG-E8	Progranulin
Fetuin A	MIG	Renin 1
Fractalkine	MIP-2	TREM-1
Gp130	MIP-3b	TROY
Granzyme B	MMP-3	
Significantly changed		
Analyte	Young	Old
Adiponectin	7593.4 ± 523.3	10644.5 ± 904.8***
bFGF	1091.3 ± 231.5	2164.7 ± 327.5***
CCL6	6374.5 ± 337.8	4067.8 ± 446.8***
Fcg RIIB	10529.8 ± 2496.2	19569.5 ± 2019.8***
IL-1a	48.1 ± 6.3	95.4 ± 4.8***
JAM-A	3644.4 ± 750.9	8718.4 ± 768.8***
Chemerin	58082.5 ± 5765.8	38676.6 ± 10073.9**
Dkk-1	3668 ±1070	1283.6 ± 227.1**
Eotaxin	29.5 ± 9.6	64 ± 18.4**
Flt-3L	3257.5 ± 535.4	4710±527**
IGFBP-5	8607 ± 685.6	6690 ± 968.3**
IGFBP-6	2106.5 ± 283.1	1351.6 ± 272**
LIX	804.8 ± 198.8	1746.3 ± 324**
RANTES	82.1 ± 23.7	585.6 ± 171.3**
Resistin	1860.6 ± 672.4	4302.7 ± 1217.3**
TACI	10599.4 ± 12260.6	157233.9 ± 71367.6**
TNF RII	2699.9 ± 342.1	3440.6 ± 216.7**
TremL1	32227 ± 3780	45409.9 ± 4734.9**
VEGF R1	2532±592.1	5968.2 ± 1345.4**
ACE	32359.4 ± 6618.2	43553.3 ± 7423.8*
C5a	78.8 ± 14.7	107 ± 20.9*
CD40L	1026.9 ± 357.2	1535.7 ± 237.8*
E-Cadherin	8003.6 ± 1330.2	10267.8 ± 980.8*
E-selectin	2207.3 ± 522.1	3372.1 ± 651.5*
Endoglin	3484.8 ± 1484.7	7774.9 ± 2752.3*
Gas 1	1657.7 ± 385.3	2367.8 ± 307.8*
Gas 6	1952.9 ± 495.7	3482.9 ± 846.8*
ICAM-1	5172 ± 1733.6	8071.4 ± 1319.4*
IGFBP-3	13633.4 ± 3189	20585.3 ± 4191.9*
IL-1ra	741.2 ± 475.1	4132.3 ± 1804.1*
MDC	20.3 ± 3.5	11.5 ± 4.9*
MIP-1g	1722.3 ± 154	2016.7 ± 202.1*
MMP-2	3568.7 ± 829.9	5844.7 ± 1645.2*
Nope	1596.3 ± 148.6	1183.9 ± 306.9*
OPG	2212.2 ± 525.4	1299.2 ± 331.9*
Osteoactivin	545.4 ± 224.6	3849.7 ± 1711.6*
PDGF-AA	86.4 ± 27.1	197.5 ± 69.8*

**Extended Data Table 3:** Quantibody array-based measurements of cytokine concentration in young and old BM fluids. BM fluids isolated from young (10 week of age) and old (27-29 months of age) mice (n=5) was analyzed using Quantibody Testing Service (Raybiotech). Samples were diluted 4-fold prior to analyses. Results are mean ± S.D. and are expressed as pg/ml concentration; *p≤0.05, **p≤0.05, ***p≤0.05.

**Extended Data Table 4:** DEGs and pathway analyses of young versus old HSCs. Significance Analysis of Microarrays (SAM) was performed on young and old cells within each population to determine SAM delta scores. The top 1000 most highly DEGs for each group were collected. SAM scores for these genes were used for GSEA and overlap with Reactome gene sets was evaluated for each population. The top 5 statistically significant (FDR<0.05) overlaps for each group are in bold.

## Supplementary Material

Supplementary Information

## Data Availability

Data sets that support the findings of this study have been deposited in the Gene Expression Omnibus (GSE169162). Source data for all the figures are provided with the paper. All other data are available from the corresponding author upon reasonable request.
