# Supplementary material for: Stromal inflammation is a targetable driver of hematopoietic aging
Source: Nat Cell Biol. Author manuscript; Available in PMC 2023 Mar 4. (PMC7614279; doi:10.1038/s41556-022-01053-0)
Supplement: Supplementary Information [file EMS170801-supplement-Supplementary_Information.zip › 41556_2022_1053_MOESM1_ESM.pdf]

nature cell biology

Article

https://doi.org/10.1038/s41556-022-01053-0

Stromal niche inflammation mediated by IL-1 signalling is a targetable driver of haematopoietic ageing

In the format provided by the authors and unedited

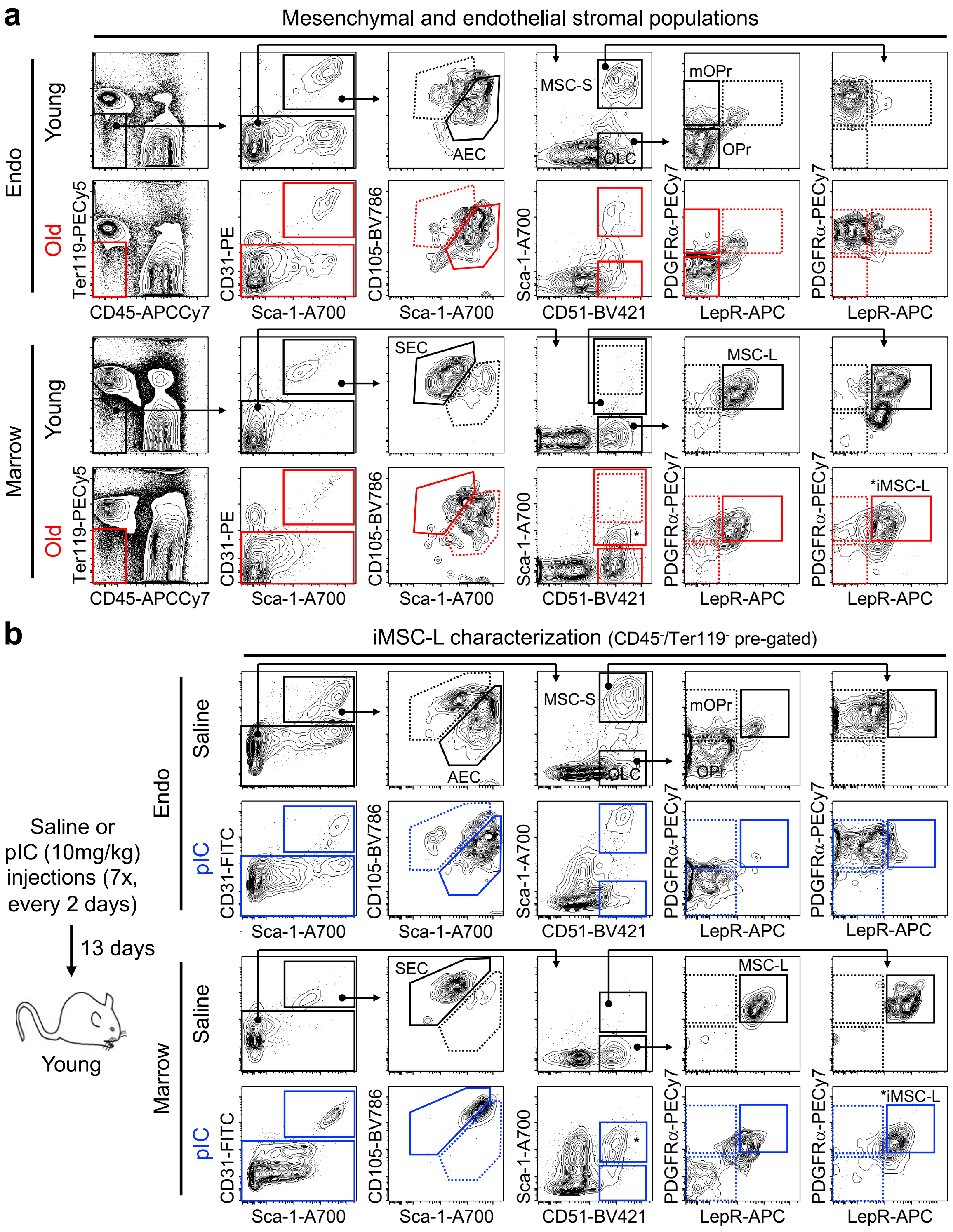

Supplementary Figure 1 | Stromal gating strategy in young and old mice. a, Representative flow cytometry staining of young and old stromal populations. AEC, arteriolar endothelial cell; SEC, sinusoidal endothelial cell; MSC-S, Sca-1^{+} mesenchymal stromal cell; OLC, osteolineage cells; mOPr, multipotent osteoprogenitor; OPr, osteoprogenitor; MSC-L, LepR^{+} mesenchymal stromal cell; iMSC-L, inflammatory Sca-1^{low} MSC-L (star). **b**, Induction of central marrow iMSC-L (star) upon injection of synthetic polyinosinic-polycytidylic acid double-stranded RNA (pIC) in young mice with experimental scheme (left) and representative flow cytometry staining of endosteal and central marrow populations (right).

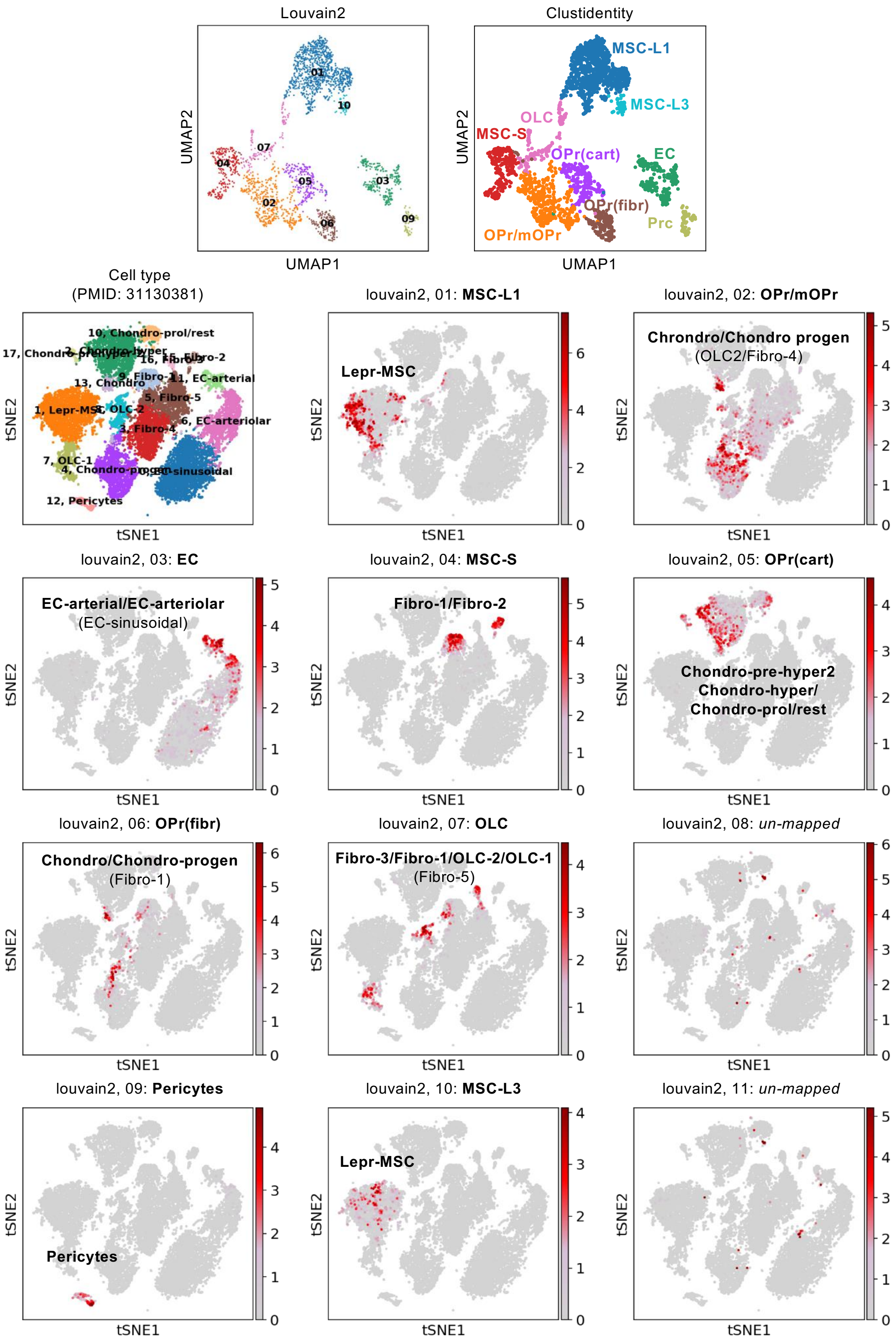

Supplementary Figure 2 | Annotation of wild type stromal droplet-based scRNAseq dataset. Cluster identification of stromal populations (Fig. 2c) based on projection into PMID: 31130381 reference UMAP[13].

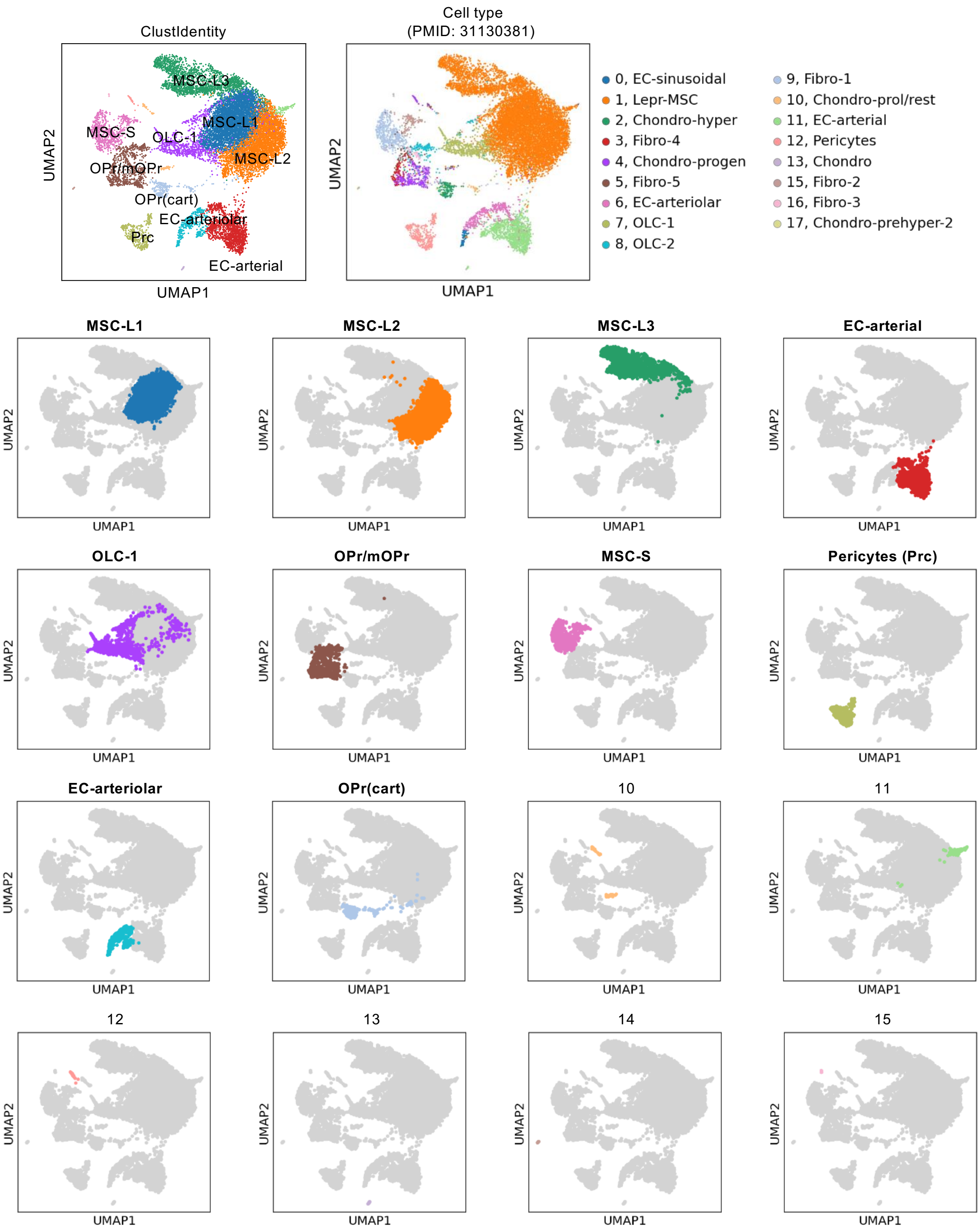

Supplementary Figure 3 | Annotation of *Il1r1*$^{-/-}$ stromal droplet-based scRNAseq datasets. Cluster identification of stromal populations (Fig. 7b) with datasets normalized using PMID: 31130381 as reference and ClustIdentity based on the most abundant/frequent reference cell type.

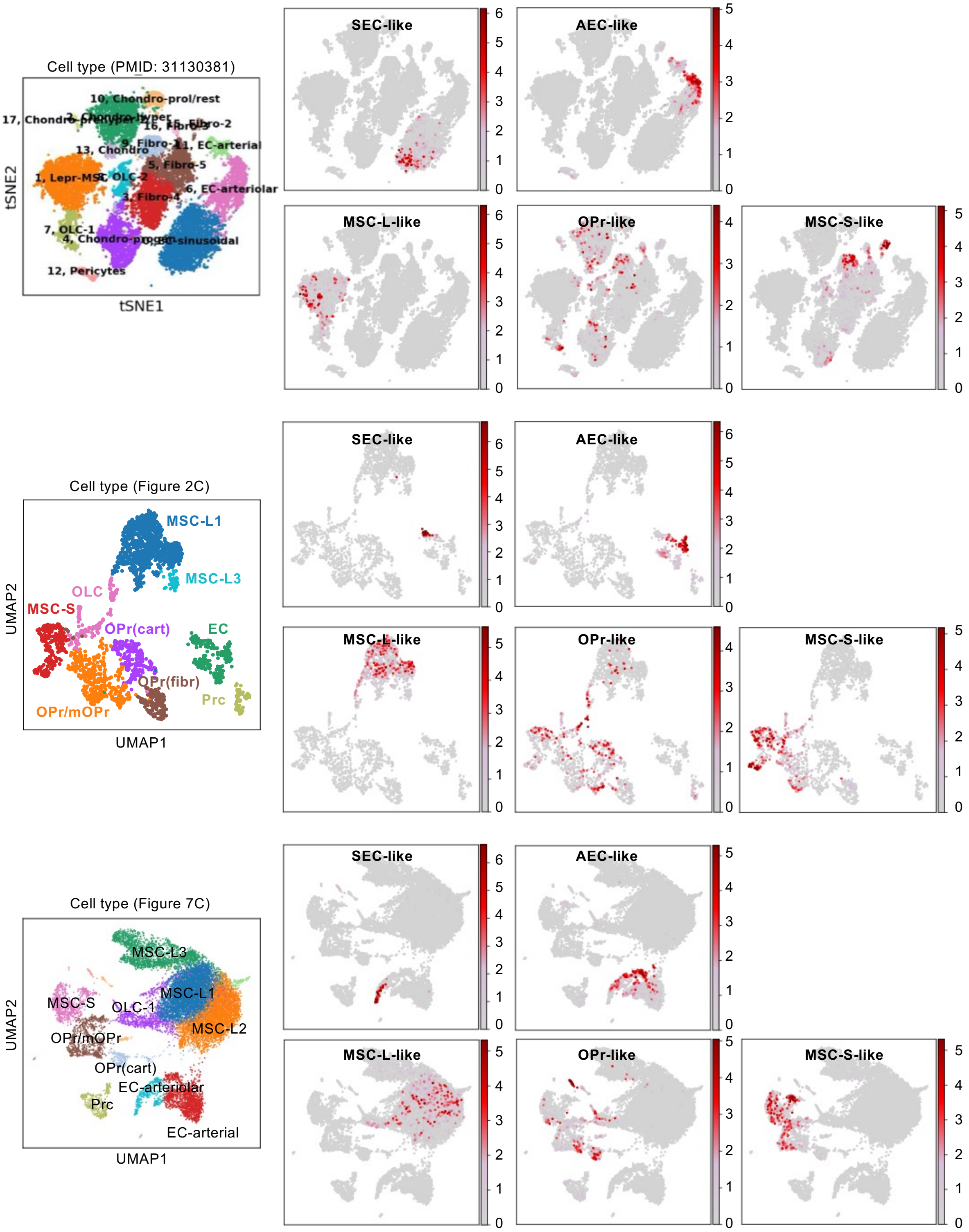

Supplementary Figure 4 | Annotation of wild type stromal plate-based scRNAseq dataset. Plate-based and droplet-based scRNA-seq data integration confirming gene identification of isolated stromal populations.

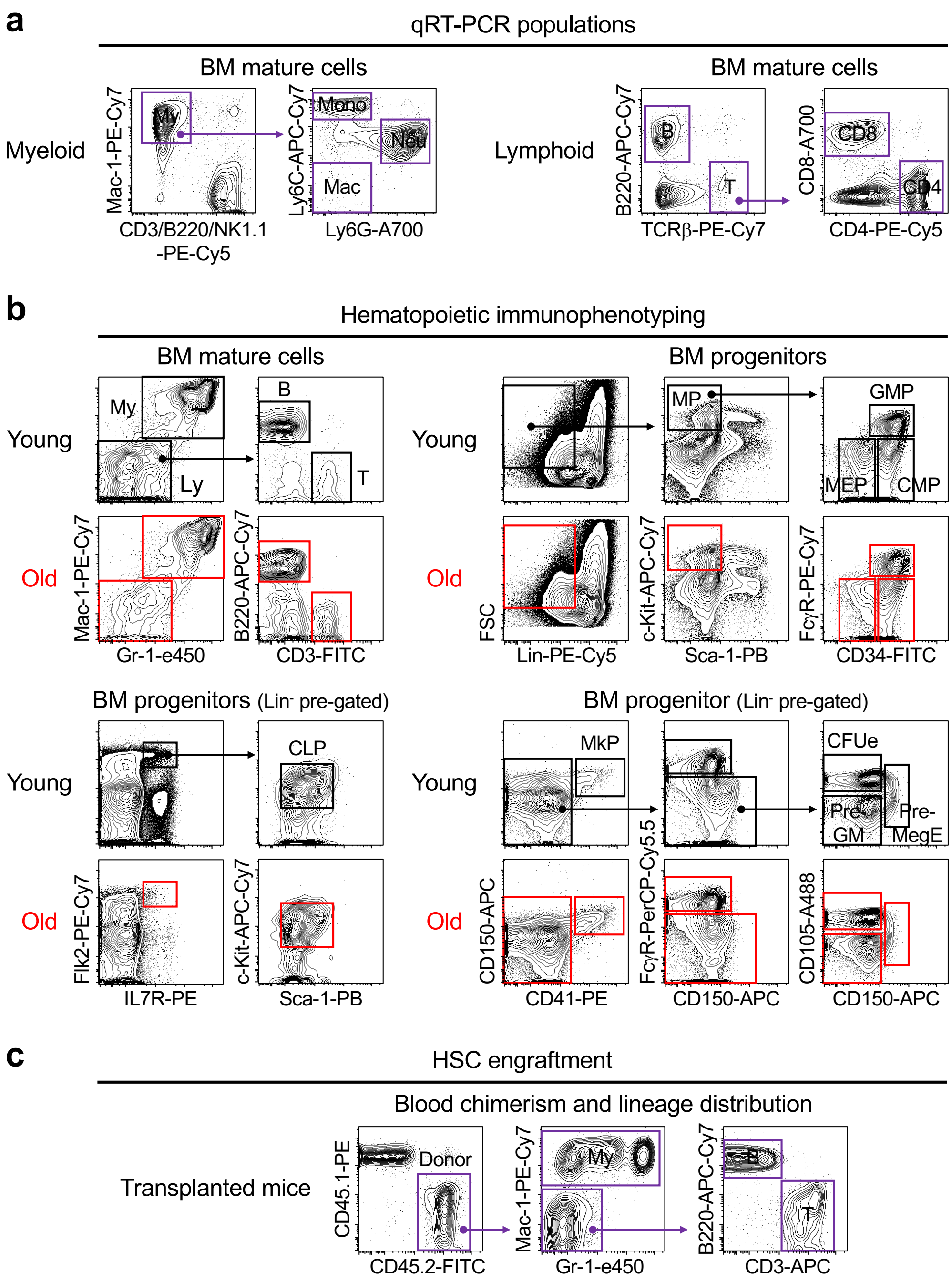

Supplementary Figure 5 | Hematopoietic gating strategy in young and old mice. a, Representative flow cytometry staining for isolating the indicated mature hematopoietic populations for qRT-PCR analyses. My: myeloid cells; Mono, monocytes; Neu, neutophils, Mac, macrophages; B, B cells, T, T cells. **b**, Representative flow cytometry staining of young and old BM cells with gating strategy for mature myeloid (My) and lymphoid (Ly) cells (top left), myeloid progenitors (top right), lymphoid progenitors (bottom left), and Mk and erythroid progenitors (bottom right). CMP, common myeloid progenitor; GMP, granulocyte-macrophage progenitor; MEP, megakaryocyte-erythrocyte progenitor; CLP, common lymphoid progenitor; MkP, megakaryocyte progenitor; Pre-GM, pre-granulocyte/macrophage; Pre-MegE, pre-megakaryocyte/erythrocyte; CFU-E, erythroid colony-forming unit. **c**, Representative flow cytometry staining of blood chimerism and lineage distribution at 4 months post-transplantation in CD45.1 recipient mice engrafted with CD45.2 donor HSCs.

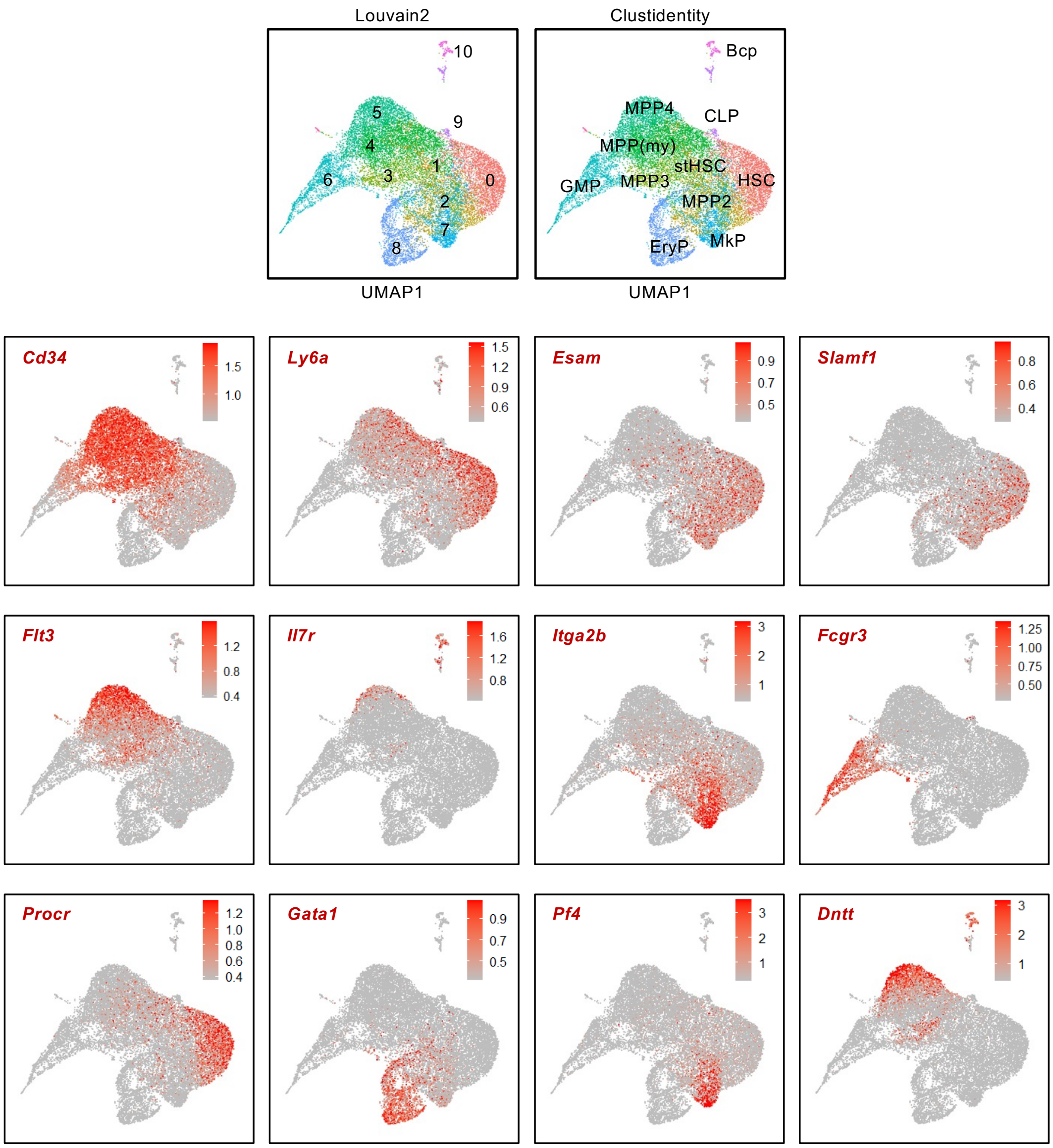

Supplementary Figure 6 | Annotation of hematopoietic droplet-based scRNAseq datasets. Cluster identification of hematopoietic stem and progenitor populations (Fig. 4b) based on expression of known ID genes[44,45].
